# Epigenetics and autoimmune diseases: the X chromosome-nucleolus nexus

**DOI:** 10.3389/fgene.2015.00022

**Published:** 2015-02-16

**Authors:** Wesley H. Brooks, Yves Renaudineau

**Affiliations:** ^1^Department of Chemistry, University of South FloridaTampa, FL, USA; ^2^Research Unit INSERM ERI29/EA2216, SFR ScinBios, Labex Igo “Immunotherapy Graft, Oncology”, Réseau Épigénétique et Réseau Canaux Ioniques du Cancéropole Grand Ouest, European University of BrittanyBrest, France; ^3^Laboratory of Immunology and Immunotherapy, Hôpital MorvanBrest, France

**Keywords:** X chromosome, polyamines, nucleolus, NETosis, autoimmune disease, epigenetics, lupus, Alu

## Abstract

Autoimmune diseases occur more often in females, suggesting a key role for the X chromosome. X chromosome inactivation, a major epigenetic feature in female cells that provides dosage compensation of X-linked genes to avoid overexpression, presents special vulnerabilities that can contribute to the disease process. Disruption of X inactivation can result in loss of dosage compensation with expression from previously sequestered genes, imbalance of gene products, and altered endogenous material out of normal epigenetic context. In addition, the human X has significant differences compared to other species and these differences can contribute to the frequency and intensity of the autoimmune disease in humans as well as the types of autoantigens encountered. Here a link is demonstrated between autoimmune diseases, such as systemic lupus erythematosus, and the X chromosome by discussing cases in which typically non-autoimmune disorders complicated with X chromosome abnormalities also present lupus-like symptoms. The discussion is then extended to the reported spatial and temporal associations of the inactive X chromosome with the nucleolus. When frequent episodes of cellular stress occur, the inactive X chromosome may be disrupted and inadvertently become involved in the nucleolar stress response. Development of autoantigens, many of which are at least transiently components of the nucleolus, is then described. Polyamines, which aid in nucleoprotein complex assembly in the nucleolus, increase further during cell stress, and appear to have an important role in the autoimmune disease process. Autoantigenic endogenous material can potentially be stabilized by polyamines. This presents a new paradigm for autoimmune diseases: that many are antigen-driven and the autoantigens originate from altered endogenous material due to episodes of cellular stress that disrupt epigenetic control. This suggests that epigenetics and the X chromosome are important aspects of autoimmune diseases.

## INTRODUCTION – EPIGENETICS AS CHROMATIN DYNAMICS

Epigenetics is the area of biology that connects environmental factors with gene expression patterns in cells. We are still discovering new aspects of epigenetics so a detailed and comprehensive definition of epigenetics is still developing (Bird, 2007; Berger et al., 2009). There is complexity to epigenetics and its dynamics since the scope of epigenetics ranges physically from individual DNA base pairs (bp) to chromosomes and ranges temporally from individual steps of the cell cycle in somatic cells to generational inheritance of gene expression patterns from parent to child. As a general definition we can consider epigenetics to be a means by which expression levels of genes and the resulting RNA and protein levels originating from the genes, can be controlled without alteration of the DNA sequence of the gene. Epigenetic regulation of gene expression includes heritable and reversible DNA modifications, modifications of DNA-binding proteins, such as histones, and generation of microRNAs (miRNAs) that interact with messenger RNAs (mRNAs) leading to degradation of the mRNA thereby suppressing gene products (Chuang and Jones, 2007). And we need to consider epigenetics as dynamic since gene expression patterns under epigenetic control can change in development, differentiation, and in response to cellular stress.

The DNA methylation status in chromatin is a key feature in epigenetic control. Methylation of carbon 5 in cytosine rings (^5m^C) in eukaryotes, particularly in promoter regions of genes, is associated with suppression of gene expression since the ^5m^C alters protein binding sites and recruits additional chromatin modifying factors, such as histone deacetylases. This control is supported by binding of proteins, such as the DNA methyl binding protein 2 (MECP2), which add another layer of control, supporting suppression or activation depending on the transcriptional context of the underlying genes. In the case of MECP2, it can bind both ^5m^C and 5-hydroxymethylcytosine (^5hm^C). However, DNA methylation can be reversed by a stepwise process that involves the recently discovered conversion of ^5m^C to ^5hm^C, followed by conversion to unmethylated cytosine. This process, ^5m^C to ^5hm^C to cytosine, is not yet fully understood but reversal of DNA methylation can potentially lead to changes in the expression of the underlying genes (Pfeifer et al., 2013).

Another important feature of epigenetic control is the packaging and compaction of DNA by histones. The basic unit of chromatin is the nucleosome which consists of approximately 145 bp of DNA wrapped around an octameric core of histones. Most DNA is associated with nucleosomes which occur on average approximately every 200 bp in humans. The cationic charges of arginine and lysine residues in the histones counter the self-repulsion of the anionic DNA allowing for compaction of the chromatin, making the underlying gene less accessible. However, post-translational modification of histones, such as acetylation or methylation of arginine residues in the histone, can reduce cationic charges on the histones and loosen the histone-DNA interactions in the nucleosome, contributing toward greater access to the underlying gene. These epigenetic modifications are reversible which can then contribute toward histone–DNA interactions that alter accessibility to the underlying gene. In addition, subnucleosomal complexes, such as histone hexamers bound to DNA, asymmetric histone modifications in the nucleosome, and histone subtypes give even more variation in the accessibility and control of genes (Rhee et al., 2014). The nucleosomes and DNA can appear as “beads on a string” when the chromatin is most accessible, which is referred to as euchromatin. Euchromatin is considered to be areas of chromatin that are transcriptionally active or at least potentiated for activity. When the nucleosomes are stacked together facilitated by histone H1 which binds to the linker DNA between nucleosomes, the DNA is less accessible, and appears to be predominantly inactive. This dense packing of the DNA and nucleosomes is referred to as heterochromatin.

Another group of factors in epigenetics is the miRNAs (Ha and Kim, 2014). miRNAs serve as post-transcriptional regulators of an estimated one-third of mRNAs. miRNAs are known to be involved in regulating apoptosis, cellular differentiation, cell cycling, and immune functions (Pauley et al., 2009). miRNAs are first transcribed by RNA polymerase II yielding primary miRNAs (pri-miRNAs; Lee et al., 2004). The pri-miRNA is processed by Drosha and Dicer in the nucleus to yield an approximately 70 base precursor miRNA (pre-miRNA) which is exported to the cytoplasm (Denli et al., 2004). Dicer then cleaves the pre-miRNA to a miRNA of 19–25 bases, which is loaded into the RNA-induced silencing complex (RISC). This complex can bind the target mRNAs and facilitate degradation of the mRNA, post-transcriptionally suppressing the gene expression (Ceribelli et al., 2011). Multiple miRNAs can target a specific mRNA and some individual miRNAs can target multiple mRNAs.

Long non-coding RNAs (lncRNAs), which can be 100s–1000s of bases in length, also have roles in epigenetic control. The lncRNAs can suppress transcription from multiple genes. The X-inactivation specific transcript (XIST) is an example of the lncRNAs. XIST RNA is involved in the silencing of one of the two X chromosomes in female cells. Since most X-linked genes are used at equivalent levels in both male and female cells, only one X chromosome is needed. Early in embryonic development each human female cell randomly chooses one of its two X chromosomes to be inactivated, leaving only one active X chromosome. This establishes X-linked gene dosage compensation such that female and male cells have equivalent expression and product levels for most X-linked genes. The X chromosome selected for inactivation expresses multiple copies of XIST which bind to contiguous chromatin along the X chromosome and recruit other epigenetic suppressing factors, such as DNA methyltransferases (DNMTs). X inactivation results from synergy of XIST RNA, DNA methylation, histone deacetylation as well as many other factors (Csankovszki et al., 2001). The result is that 75–85% of genes on the inactive X chromosome are silenced or have reduced expression relative to the active X chromosome (Carrel and Willard, 2005; Cotton et al., 2013). The X-linked genes that show variable escape or reactivation from X inactivation, approximately 5% of genes on the long arm Xq and 35% on the short arm, Xp, are more often located close to or even between genes that are normally expressed from both the active and inactive X chromosomes, such as genes at Xp22.1 on the short arm (Carrel and Willard, 1999; Carrel et al., 1999). Daughter cells will inherit the same X inactivation patterns although there can be infrequent reactivation of some genes on the inactive X with age (Wareham et al., 1987), during some stages of development (Ohhata and Wutz, 2013), or as a result of chemical insult, such as with the demethylating agent 5-azacytidine (Venolia et al., 1982). The genes that escape from X inactivation can vary among cell lines and tissue types (Carrel and Willard, 2005). The possibility of reactivation of X-linked genes in somatic cells is an area of current interest since it may have an underlying role in some disease mechanisms. Duplication and/or reactivation of X-linked genes and even X chromosomes has been reported in some tumors and is infrequently observed in cell cultures (Ohhata and Wutz, 2013). We should note that: (1) most studies on X inactivation and X-linked gene reactivation from the inactive X chromosome are performed with mouse cells or human-mouse hybrid cells; (2) the mouse X chromosome has proven to be problematic in these studies since the mouse X chromosome is very robust in absorbing experimental chemical insults applied to study the stepwise reactivation of individual genes of the inactive X, i.e., partial reactivation of the inactive X; (3) these studies monitor protein coding genes transcribed by RNA polymerase II (RNA pol II) whereas non-coding X-linked genes and elements transcribed by X-linked RNA polymerase III (RNA pol III) may be more informative, as discussed below, since their transcriptional activation is not as complicated as that of RNA pol II transcribed genes.

We can view epigenetics as a dynamic process since the reversible nature of epigenetic control allows for changes that can open silenced genes to become potentially active genes and back. And it can convert large regions of chromatin from euchromatin to heterochromatin and back. A recent report describes the three-dimensional arrangement of the human genome at a resolution of 1 kb (Rao et al., 2014). The authors reported that the human genome structure has approximately 10,000 chromatin loops most of which were less than 2 mb with the majority of the loops anchored by the transcriptional regulator CCCTC-binding factor (CTCF). These loops can provide more accessibility to the genes in the loop and allow for dynamic inter-loop associations of genes bringing them into a shared context. The authors also reported interesting observations on the inactive X chromosome structure, that it had two major domains and it had six super loops, four of which were associated with the sites of lncRNA genes (*DXZ4*, *XIST*, *loc550643*, and *FIRRE*). Each of these lncRNAs may have a role in maintenance of the X inactivation state in their vicinity. The two domains observed in the inactive X chromosome lay on either side of the *DXZ4* gene, which is in the middle of the X long arm, Xq (Chadwick, 2008). These domains and lncRNAs suggest bipartite, even multipartite aspects to the epigenetic control of the genes and regions of the inactive X chromosome.

The involvement of epigenetics in autoimmune disorders has become a topic of increasing interest as reports accumulate of epigenetic dysregulation associated with specific autoimmune disorders (Brooks et al., 2010; Thabet et al., 2013; Konsta et al., 2014). Epigenetic dysregulation due to methylation/demethylation has been reported in regards to specific genes and autoimmune disorders, such as inability in some lupus patient B lymphocytes to methylate the promoter of the human endogenous retrovirus (HERV) gene, *HRES1/p28*, leading to its overexpression in lupus (Fali et al., 2014). Another example is the expression of the CD5 protein in B lymphocytes. CD5 is a cell surface protein involved in intracellular signaling to suppress autoreactivity. In some lupus patients, an alternative promoter becomes demethylated leading to a switch from the CD5-E1A isoform normally found at the cell surface to the CD5-E1B isoform that is retained in the cytoplasm resulting in a failure to suppress autoreactivity (Garaud et al., 2009).

In reality, epigenetics is more than just the methylation state and accessibility of specific genes. Higher levels of epigenetic control mechanisms, such as X inactivation, can affect entire chromosomes containing a diverse collection of genes. Epigenetics also involves spatial relationships of genes that are brought into close proximity for a common purpose (e.g., genes for enzymes in a common pathway) so that their expression can be more efficiently regulated. Epigenetic suppression and compacting of chromatin also stores potential DNA supercoiling stress that, when released, can disrupt and alter chromatin over hundreds even thousands of bps, rapidly unraveling heterochromatin into more accessible extended loops. Each nucleosome stores supercoiling stress that, when released, can flux through the chromatin causing twisting and disruption of the chromatin structure as the chromatin adjusts to accommodate the stress (Brooks, 2013). The released supercoiling stress can also allow the transient appearance of alternate DNA conformations which can mask protein binding sites and slow repair and replication of DNA. In this manner we can envision potential temporal effects in cells, such as delays in S phase replication of some chromatin when there is epigenetic dysregulation. In the extreme, there could be loss of genes and disproportionate inheritance of genetic material by daughter cells with epigenetic dysregulation. Thus, epigenetic control must be maintained even during cellular stress. We can think of epigenetics as the dynamics of chromatin that occurs at many levels from short stretches of DNA capable of flipping to alternate conformations, to nucleosomes, to loops of hundreds of nucleosomes, to entire chromosomes. And we should think of the spatial and temporal effects of epigenetics since epigenetic changes can alter the timing and success of chromatin replication, repair, and daughter cell inheritance.

## THE FEMALE PREDOMINANCE OF AUTOIMMUNE DISEASES

**FIGURE 1:**
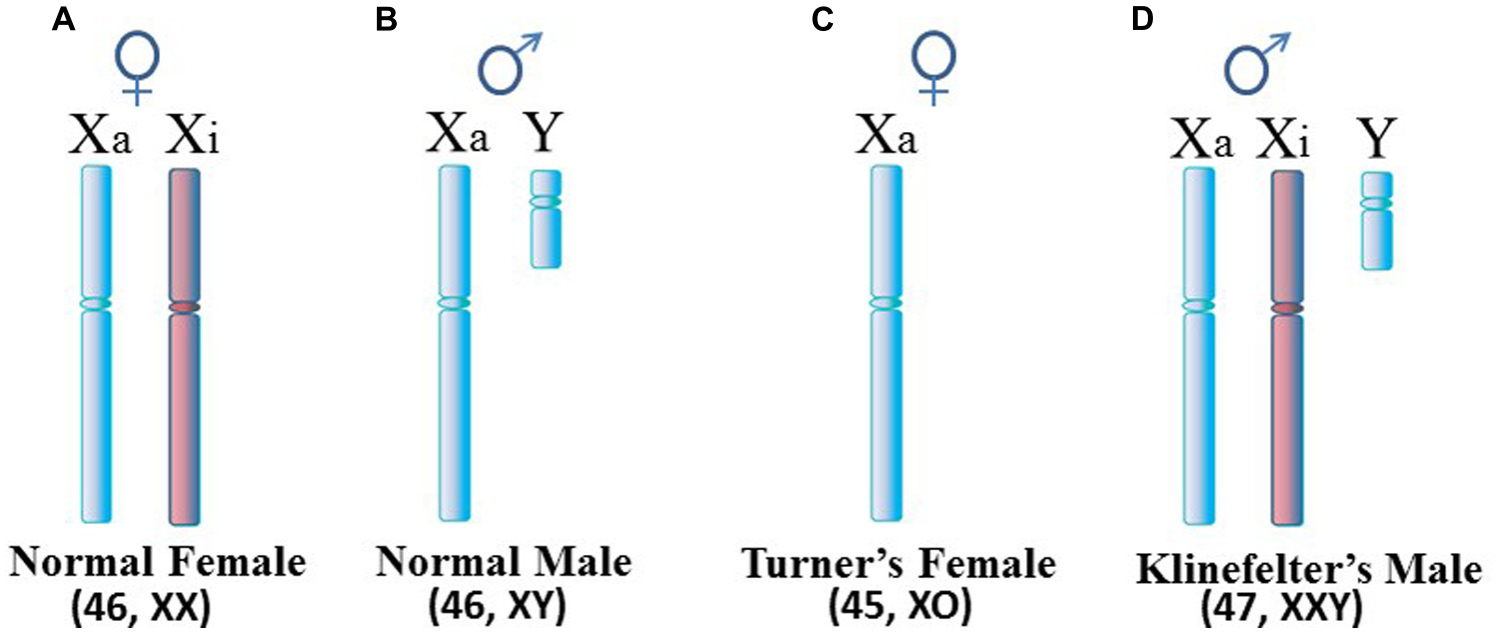
**Human sex chromosomes. (A)** Normal female cells contain an active X (Xa) and an inactive X (Xi). Since most X-linked genes are not sex-specific, female cells really only need expression from one X, similar to **(B)** the normal male. **(C)** Turner’s syndrome females typically have only one X per cell but variations can occur. **(D)** Klinefelter’s syndrome males have an extra X which is usually inactivated. For X inactivation to occur, it requires at least two X inactivation centers (XICs) in close proximity so that a stoichiometric-triggered random selection can be made as to which X to inactivate, the maternally derived X or paternally derived X. Each daughter cell will maintain inactivation of the same parentally derived X thereafter.

Autoimmune diseases are estimated to affect 5–10% of the population with the majority of autoimmune disease patients being female. However, the female:male ratio differs among the diseases. For example, the ratio is only slightly above 1:1 in inflammatory bowel disease and diabetes mellitus type 1, whereas the ratio is approximately 2:1 in multiple sclerosis (MS), 3:1 in rheumatoid arthritis (RA), and 9:1 to 10:1 in systemic lupus erythematosus (SLE), Sjögren’s syndrome (SjS) and autoimmune thyroiditis (Invernizzi et al., 2009). This overall female predominance suggests possible involvement of the X chromosome. Since females normally have two X chromosomes (**Figure 1A**) in each cell while male cells have only one X chromosome (**Figure 1B**), this suspicion of the X is supported by the observed rates of SLE in Klinefelter’s syndrome in which (47, XXY) males with an extra X chromosome have a rate of SLE 14x greater than (46, XY) males (**Figure 1D**; Scofield et al., 2008; Sawalha et al., 2009). On the other hand, the relationship between the X chromosome and autoimmune diseases appears to be more nuanced when one considers Turner’s syndrome and autoimmune diseases. Turner’s syndrome is typically thought of as an X monosomy (45, XO) female (**Figure 1C**). Previously it was thought that rates of autoimmune diseases in Turner’s syndrome patients would be similar to rates observed in (46, XY) males, in effect, a rate lower than seen in (46, XX) females. In reality, there are no published reports of classic (45, XO) Turner’s syndrome with SLE, possibly due to a lack of specific studies to address this question (Scofield et al., 2008). However, Turner’s syndrome symptoms can occur in a range of karyotypes, such as a mosaic (45, XO; 46, XX) female in which some cells are XO and some are XX, or other situations in which only a portion of the second X is present. SLE has been reported recently in a Turner’s syndrome patient with a [46, XX del(Xq13-ter)] karyotype in which there is one complete X chromosome but much of the long arm of the second X chromosome is missing (Cooney et al., 2009). There is a need for more analysis of the relation between Turner’s syndrome and autoimmune diseases to assess the frequency of co-occurrence and to decipher which variations in the Turner’s syndrome scenario contribute to the autoimmune disease symptoms. Toward this objective, a recent study on a cohort of 798 Turner’s syndrome patients in Denmark reported a rate of autoimmune disease comorbidity with Turner’s syndrome at approximately double the rate for females of (46, XX) karyotype, but the rate of predominantly female autoimmune diseases (e.g., Hashimoto’s thyroiditis) was 1.7x higher in Turner’s syndrome females versus (46, XX) females (Jørgensen et al., 2010). For other autoimmune diseases that typically have a slight male predominance (e.g., Type 1 diabetes), the Turner’s syndrome patients had a 5x higher rate than (46, XX) females. The study did not report any cases of Turner’s syndrome with SLE among the patients and only three cases of Turner’s syndrome with RA.

This general approach of analyzing the infrequent occurrences of autoimmune symptoms in what might otherwise be considered non-autoimmune disorders can be very beneficial in expanding our understanding of autoimmune diseases. But we must be open to broad explanations that can involve genetics and/or epigenetics, and can involve the innate immune response, the adaptive immune response, and even events preceding any immune involvement.

## AUTOIMMUNE DISEASES AND GENE SEQUENCES OF THE X CHROMOSOME

**FIGURE 2:**
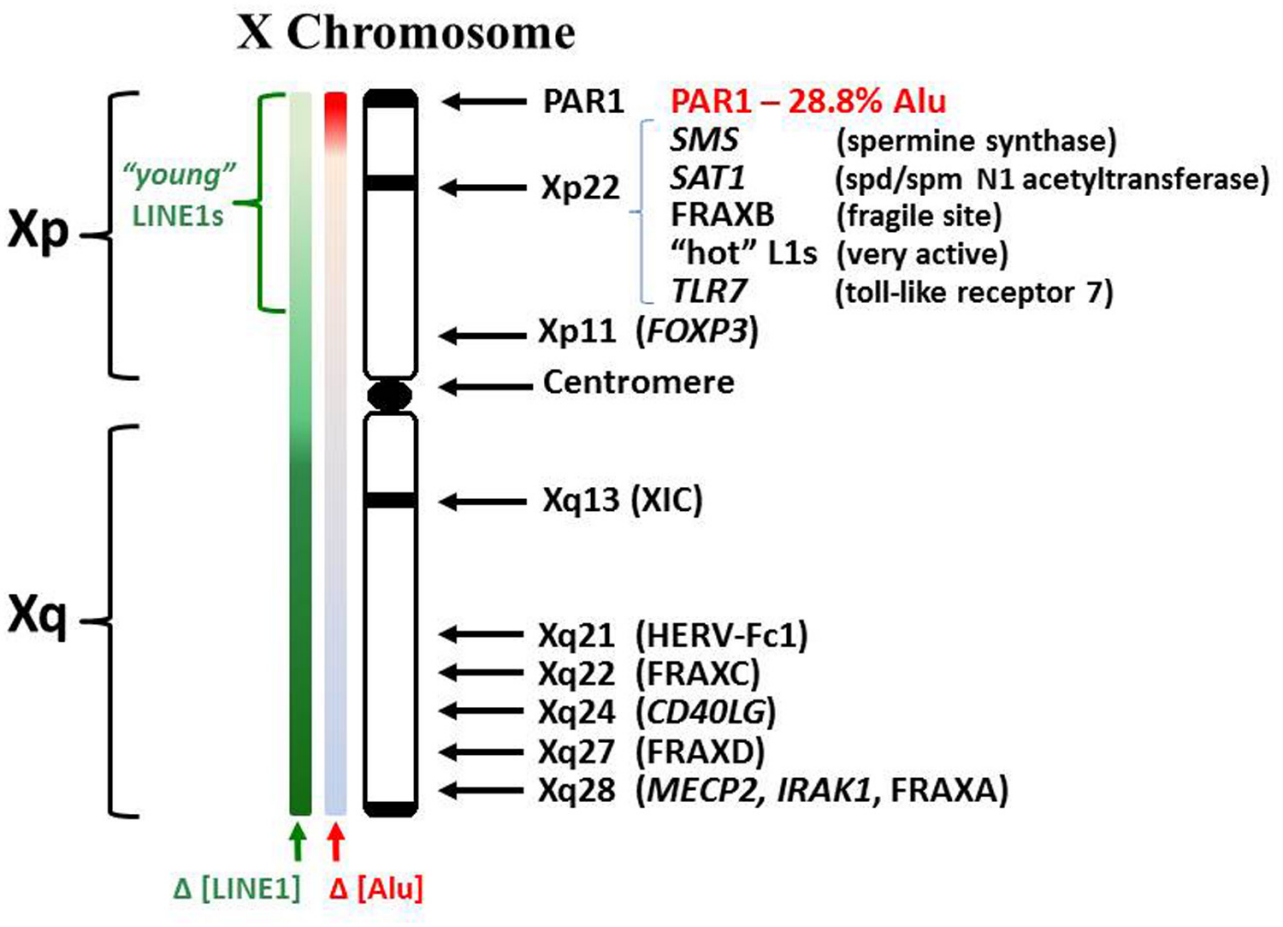
**Human X chromosome features.** The human X consists of a short (Xp) and long (Xq) arm separated by a centromere. The XIC expresses the X inactivation-specific transcript RNA (XIST RNA) which binds contiguous chromatin and recruits enzymes involved in establishing epigenetic silencing. LINE1 elements (L1) serve as anchoring sites for XIST RNA. Whereas L1 comprises 17% of the human genome, it comprises 34% of the X chromosome (Ross et al., 2005). L1 content drops in Xp but consists of more recently incorporated L1s, some still capable of reverse transcription. Lower L1 content in Xp suggests potential difficulties in maintaining Xp inactivation. Alu elements comprise ~10.8% of the human genome but only 8% of the X. However, the PAR1 region is 28.8% Alu. Alus contain an internal RNA pol III transcription site but are usually suppressed by a positioned nucleosome.

In relation to the X chromosome, a few specific X-linked genes have demonstrated an association with autoimmune diseases (**Figure 2**). In some cases, the gene in question may have genetic mutations, insertions, deletions, or duplications that alter the gene product function and/or the levels of gene expression. In other cases there might be epigenetic changes that alter the level of gene expression without changes in the underlying DNA sequence. The methyl CpG binding protein (MECP2), which suppresses transcription by capping methylated DNA sites and recruiting histone deacetylases, shows decreased *MECP2* mRNA in association with its contributing risk for lupus (Kaufman et al., 2012; Sawalha, 2013). This decreased *MECP2* mRNA expression appears to result from single nucleotide polymorphisms (SNPs) in the vicinity of the *MECP2* gene, SNPs that alter the gene expression but not the functioning of the protein. The *MECP2* gene is located at Xq28, i.e., toward the end of the long arm of the X chromosome.

Another gene that has been linked to autoimmune diseases is the interleukin-1 receptor-associated kinase 1 gene (*IRAK1*) which is also located at Xq28 near the *MECP2* gene. IRAK1 interacts with the interleukin-1 receptor to up regulate a transcription factor, nuclear factor κB activating protein (NKAP), which activates the innate immune response. Several SNPs have been identified in the DNA sequence in and around *IRAK1*. One mutation in the gene leads to an S196F change in the IRAK1 protein which leads to enhanced up regulation of NKAP and, thereby, contributes to an increased risk for lupus (Kaufman et al., 2012).

Another gene linked to autoimmune diseases is *CD40LG* (a.k.a. *CD154*), located at Xq24, which codes for a membrane protein expressed at the surface of activated CD4 T cells (Banchereau et al., 1994). CD40LG binds the costimulatory receptor CD40 on antigen presenting cells, such as macrophages. This CD40–CD40LG interaction then begins activation of an adaptive immune response toward the antigen. In systemic sclerosis (SSc) *CD40LG* is overexpressed, which sets up an overly sensitive response (Lian et al., 2012). The mechanism of this *CD40LG* overexpression is not yet understood. However, epigenetic dysregulation is suspected since demethylation of *CD40LG* on the inactive X chromosome of T cells in lupus patients has been reported (Lu et al., 2007).

FOXP3 (forkhead box P3), also believed to have involvement in some autoimmune diseases, is a key transcription factor controlling activation of regulatory T cells (Treg cells). The methylation status of the gene *FOXP3*, which is located at Xp11.23, helps determine its expression which, in turn, induces other important genes in the Treg cells, such as the T cell receptor (Kim and Leonard, 2007).

Human endogenous retrovirus are suspected of roles, possibly reverse transcription activity, in autoimmune diseases (Le Dantec et al., 2012; Nissen et al., 2013). Among these, *HERV-Fc1*, with only one copy in the genome located at Xq21.33, has shown increased expression in cases of MS (Nissen et al., 2012, 2013; de la Hera et al., 2014). The means by which the increased expression of *HERV-Fc1* occurs is not known but the occurrence of reverse transcription may be detrimental for the cell since the reverse transcribed DNA would be out of normal epigenetic context (e.g., not properly methylated).

Another source of potential reverse transcription activity is the long interspersed element, LINE1 (L1). L1 elements originated from a functional gene that codes for reverse transcriptase activity (RNA to DNA) and endonuclease activity that aids in inserting reverse transcribed DNA into the genome at new sites. L1 elements comprise 17% of the human genome and there are estimated to be more than 500,000 L1 element copies scattered throughout the human genome (Crow, 2010; Rodic and Burns, 2013). A definite number is difficult to obtain since most L1 element copies have undergone mutations, particularly in the 5′ region, that alter their sequence and disrupt their functionality in retrotransposition activity. Often newer copies have inserted over old copies.

Although most L1 elements have degenerated over time and lost their function as reverse transcriptases, 80–100 copies are believed to still be competent for retrotransposition activity (Alves et al., 2000). Brouha et al. (2003) have mapped and analyzed most of the L1s that are believed to be functional. They observed approximately half of these viable L1 elements have varying degrees of activity in cell cultures. *In vivo*, most L1 elements are sufficiently methylated to keep them inactive. However, recent reports have demonstrated that, in lupus patients, some L1 elements are hypomethylated in CD4+ and CD8+ T cells, B cells, and neutrophils (Nakkuntod et al., 2011; Brooks, 2014; Sukapan et al., 2014). With hypomethylation, which usually coincides with histone deacetylation, the L1 elements could become active. This could include L1 elements with functional reverse transcriptase activity (RNA to DNA conversion) and even functional retrotransposition activity (RNA to DNA conversion and insertion into the genome). But other L1 elements that become active, even if they do not have functional reverse transcriptase activity, could become problematic since they are frequently located in other genes (intragenic location) and, in some sites, the L1 elements are anti-sense to a larger gene. Expression of these L1 elements could present alternate transcription sites disrupting normal expression of the larger gene or anti-sense L1 element expression could create ssRNA that hybridizes with the larger gene’s transcripts leading to dsRNA degradation and dampening of the larger gene’s effects.

LINE1 elements are proposed to serve an additional function with regards to the X chromosome besides their retrotransposition activities, their potential as alternate transcription start sites, and their potential as anti-sense interfering RNAs. There is an overall twofold enrichment of L1 elements on the X chromosome (34%) compared to the genome average (17%) but the enrichment is in a gradient, higher in the X chromosome long arm (Xq) but it becomes lower in the short arm (Xp; Ross et al., 2005). The L1 elements on the X chromosome are believed to function as anchoring sites for the X-inactivation specific transcript RNA (XIST RNA) that is expressed from the X inactivation center (XIC) at Xq13 and spreads over contiguous chromatin in one of the two X chromosomes to silence genes and create the inactive X chromosome (Xi). This is the basis of the Lyon repeat hypothesis (Lyon, 2006). Lyon proposed that the XIST RNA anchors at the repetitive L1 elements and recruits DNMTs and histone deacetylases to instill epigenetic silencing of the underlying genes in the Xi. When X inactivation is being established initially in early development of the embryo, L1 elements help with X inactivation by forming compartments that sequester the inactive genes of the Xi into a dense core while transcription occurs from young L1 elements at the surface of this core in an attempt to extend the silencing further into areas of genes that can sometimes escape inactivation (Chow et al., 2010). The final result is silencing of approximately 75–85% of the genes on the Xi but the intensity of silencing along the X decreases in parallel with the density of L1 elements, which is lower in the X short arm (Xp) compared to the X long arm (Xq; Carrel and Willard, 2005; Lyon, 2006). The resulting Xi, (a.k.a. the Barr body) has a dense perinuclear appearance with a core of inactive genes and genes that escape inactivation at the surface. Once established, the X inactivation state is maintained throughout the cell cycle and is inherited by daughter cells. However, cellular stresses can interfere with maintenance of the DNA methylation on the Xi potentially allowing reactivation and expression of X-linked genes from the Xi. The Xi with its perinuclear location, dense packaging, and heavy requirement for methyl donors (*S*-adenosylmethionine, SAM) is the last chromatin replicated in S phase which adds to the difficulties in maintaining the properly silenced epigenetic state.

The X and Y chromosomes are believed to have originated from a common autosome. Since then, the Y has decreased to approximately 60 × 10^6^ bps and contains approximately 100 genes including the SRY sex-determining gene. The X chromosome has actually gained genes increasing to 153 × 10^6^ bps and approximately 1,100 genes. Much of the newer material is located in the Xp (Ross et al., 2005). L1 elements in the Xp are considered to be younger copies (i.e., fewer mutations) of which some are suspected of reverse transcriptase functionality. Indeed, the work of Brouha et al. (2003), mentioned above, identified ‘hot L1’ elements, in effect, those L1 elements containing complete functional sequences for the L1 reverse transcriptase and which demonstrate some occasional expression activity. Among these, the Ta-1d subclass is one of the more active and frequently occurring L1 elements with reverse transcriptase activity. At least two such sites of ‘hot L1’ elements were identified in the Xp by Brouha et al. (2003) and this includes one site of particularly strong activity at Xp22 as determined from the Blast sequence AC004554 (http://www.ncbi.nlm.nih.gov/nuccore/AC004554.1; Myers et al., 2002). Experimental activation of retroviral sequences by UVB has been reported previously (Hohenadl et al., 1999) and, in fact, expression of L1 elements was observed in RA (Neidhart et al., 2000). Even earlier, retroviral activity had been proposed for autoimmunity, including SLE (Herrmann et al., 1996; Nakagawa and Harrison, 1996). Recently, more details have been discerned that have begun the association of particular active retroviral sequences with specific diseases (Hancks and Kazazian, 2012).

It is not simply that reverse transcription could occur, but which RNA sequences are reverse transcribed, and when and where in the cell the reverse transcription occurs that are important factors with regards to involvement of reverse transcription in autoimmune diseases. Reverse transcribed DNA sequences that are rich in CpG content will require extensive *de novo* methylation. This may not be possible if the level of SAM, the cellular methyl donor, is low or if the new DNA is not located near existing DNMTs. Since most human DNMTs localize to the nucleus where they cooperate in *de novo* and maintenance methylation of DNA and only a minor amount of DNMT3a and DNMT3b is available in the cytoplasm for *de novo* methylation (Kim et al., 2002), reverse transcribed DNA created in the cytoplasm would be less likely to receive proper methylation. In lupus, the DNA targeted as autoantigenic is CpG rich and has abnormal methylation patterns, primarily hypomethylation of sequences (Krieg, 1995). In fact, the free DNA in sera of lupus patients is enriched in Alu sequences, which have a very high CpG content. Whereas Alu elements comprise approximately 10% of the human genome, the Alu content of free DNA in sera of lupus patients has been observed to be as high as 55% (Li and Steinman, 1989). One plausible explanation for this relative increase in Alu DNA is reverse transcription of Alu RNA.

Alu elements on the X chromosome present very fascinating potential with regards to autoimmune disease mechanisms. Alu elements comprise only 8% of the X chromosome, i.e., less than the genome average (Ross et al., 2005). However, Alu elements constitute 29% of the pseudo-autosomal region 1 (PAR1) at the end of Xp and 19% of the adjoining S5 region (Ross et al., 2005). Alu elements average 300 bps and contain an intragenic RNA pol III promoter, thus RNA pol III can create a complete Alu RNA transcript from within the Alu element DNA (i.e., no 5′ promoter needed). With reverse transcriptase activity and RNA pol III activity, there could be a rapid, even exponential, increase in both Alu DNA and Alu RNA serving as templates for each other. Normally Alu elements are kept silent with a nucleosome positioned over the RNA pol III promoter. Also, proteins have been identified that bind Alu DNA elements and selectively suppress RNA pol III transcription (Kropotov et al., 1999). However, the binding of some of these proteins is sensitive to the methylation status of the Alu DNA (Cox et al., 1998). Among the proteins that bind Alu DNA are the Ku proteins, often seen as autoantigens in SLE (Tsuchiya et al., 1998). Methylation of the CpG rich Alu elements would also contribute to their epigenetic packaging and suppression with subsequent histone deacetylation and MECP2 capping. However, in S phase as the DNA and Alu elements are replicated, there is a heavy demand on SAM for methylation and on the supply of proteins that reinforce Alu silencing. Silencing of Alu elements in the heterochromatic inactive X chromosome would be particularly problematic since the Xi replicates later than the other chromosomes and its extensive silencing requires availability of suppressing proteins and an ample supply of SAM which may be low by the time the cell enters late S phase. One potential problem that could result following inadequate Alu silencing is interference with assembly of signal recognition particles (SRP) which have Alu domains (Brooks, 2012). Alu RNA transcripts could compete with the SRP Alu domain for SRP9/14 heterodimers resulting in incomplete SRP that cannot halt ribosome translation in the cytoplasm of extracellular proteins which would normally be translated into the endoplasmic reticulum lumen but now are exposed to cytoplasmic enzymes. Another potential problem is reverse transcription of Alu RNA by functional L1 reverse transcriptases, such as from the ‘hot’ L1 sites in the Xp (Brooks, 2014). Dewannieux et al. (2003) demonstrated that L1 reverse transcriptases will preferentially reverse transcribe L1 RNA (1000x) and Alu RNA (300x) compared to other RNA transcripts (1x). The Alu RNA transcripts and reverse transcribed Alu DNA could disrupt and overwhelm the cell’s functions of methylation, translation, and translocation. There is an observed phenomenon of Alu stress response in which many Alu elements throughout the genome can suddenly be expressed due to shifting of nucleosomes during stress (Kim et al., 2001). This shifting of nucleosomes exposes intragenic RNA pol III transcription sites in the Alu elements.

Another set of genes on the X chromosome that have bearing on autoimmune diseases are the spermine synthase (*SMS*) gene and the spermidine/spermine-*N*1-acetyltransferase (*SAT1*) gene at Xp22.1. The enzymes from these genes are involved in the polyamine pathway: SMS in biosynthesis of spermine from spermidine and SAT1 in recycling of spermine to spermidine and spermidine to putrescine. These two genes can, in effect, work against each other leading to wasteful cycling through polyamine synthesis and recycling. Since polyamine synthesis uses SAM, this could adversely impact the availability of SAM needed for DNA methylation. Normally *SMS* and *SAT1* are silenced on the Xi (Carrel et al., 1999; Carrel and Willard, 2005) but repeated stresses could lead to cells in which these genes escape inactivation, particularly since they are near other genes that normally escape inactivation. *SAT1* is particularly interesting in that it can undergo superinduction (rapid increase in expression of 100x or more) in response to cellular stress and SAT1 can acetylate spermidine which can then be oxidized to putrescine (Chopra and Wallace, 1998). Recently it was reported that SAT1 and *S-*adenosylmethionine decarboxylase (AMD1) are elevated in RA synovial fibroblasts, along with putrescine which stabilizes AMD1 and is a precursor for polyamine synthesis (Karouzakis et al., 2012). The polyamine pathway competes with cellular methylation for the methyl donor SAM. Overexpression of *SAT1* and *SMS* could rapidly deplete SAM by futile polyamine synthesis and recycling.

The Toll-like receptor 7 (*TLR7*), located at Xp22.2, has recently been determined to be associated with increased risk for lupus in males in Chinese and Japanese populations when a SNP is found in the 5′ untranslated region (UTR) of the gene (Shen et al., 2010). Higher expression of *TLR7* in B cells can cause an increase in type 1 interferon activity.

There are over 2,000 processed miRNAs identified so far originating from the human genome (Yan et al., 2014). Approximately 10% originate from the X chromosome. Some miRNAs are involved in normal immune functions, such as TLR signaling, IgG class-switching, and B cell differentiation (Pauley et al., 2009). In addition, abnormal expression, processing, and/or functioning of miRNAs have been determined in autoimmune diseases, such as lupus (Tang et al., 2009; Zhao et al., 2011) and RA (Karouzakis et al., 2009). Abnormalities related to X-linked miRNAs have been proposed previously as causative in autoimmune disorders and the female predominance of autoimmune patients (Pinheiro et al., 2011).

One last category of DNA sequences in the X chromosome that should be mentioned is fragile sites. Fragile sites are classified as common (found in all or most members of a population) and rare (found in 5% or less of population). So far in the human genome 30 rare fragile sites and 89 common fragile sites have been identified (Debacker and Kooy, 2007). Fragile sites are stretches of DNA that are particularly susceptible to altered rates of DNA replication, constrictions, breaks, gaps, and viral insertions in part due to stalling and difficulties in sustaining smooth and complete replication through the stretches. This can lead to loss of genes, gaps in the chromosome, difficulty in maintaining epigenetic control or even fragmentation. Fragments that persist could then escape normal epigenetic control and perhaps lead to abnormal distribution of genes among daughter cells. Alternate DNA conformations (i.e., non-B-DNA) can occur in fragile sites and slow replication until they are resolved. We can consider the inactive X chromosome to be particularly problematic with regards to replication since it replicates late in S phase (in fact the last chromosome), it has a limited time to effect DNA repair, and it requires extensive DNA and histone methylation to attain proper repackaging. In addition, the inactive X requires more scaffold attachment factor A (SAF-A) to hold its dense perinuclear structure but SAF-A itself requires methylation in order to translocate to the nucleus (Helbig and Fackelmayer, 2003).

With regards to the X chromosome, three rare fragile sites with their locations have been identified: FRAXA (Xq27.3, associated with gene *FMR1*); FRAXE (Xq28, associated with *FMR2*); and FRAXF (Xq28, associated with gene FAM11A) and three common fragile sites have been identified: FRAXB (Xp22.31); FRAXC (Xq22.1); and FRAXD (Xq27.2; Debacker and Kooy, 2007). Associations with specific genes for the X-linked common fragile sites have not yet been made. These fragile sites typically replicate later than neighboring non-fragile alleles. For example, fragile FRAXA alleles at Xq28 replicate in the G2/M phase whereas neighboring non-fragile alleles replicate in late S phase (Hansen et al., 1993). This late replication may be attributable to formation of alternate non-B DNA structures (e.g., hairpins) within the fragile site that hamper polymerase movement and function (Usdin and Grabczyk, 2000). On the other hand, other sites, such as FRAXB, show greater flexibility in their fragile site DNA due to higher AT-rich repeats interspersed with interruptions (Arlt et al., 2002). It is conceivable that these fragile sites have difficulties during replication resolving fluxing supercoiling stress that is released. In AT-rich repeats the DNA strands may separate more readily (lower melting point) but then may form intra-strand hybridization that may hamper smooth polymerase movement. The abundant Alu elements have the capability of such cruciform formation as seen in the Alu domain of the 7SL RNA of the SRP. We should note that, fragile sites are not necessarily the same as chromosomal break points, but they often coincide or are in close proximity, such as seen at the *FMR1* gene in FRAXA (Verkerk et al., 1991).

## X CHROMOSOME ABNORMALITIES AND AUTOIMMUNE DISEASE SYMPTOMS

X-linked chromosomal abnormalities have been implicated in many disorders, such as Fragile X, Turner’s, and Klinefelter’s syndromes. As an example of possible associations of X abnormalities and autoimmune diseases, chromosomal aberrations have been reported in MS and many of those aberrations are related to the X chromosome (D’Alessandro et al., 1990). A report on the parent–child correlation in MS sheds further light on the MS and X chromosome association (Sadovnick et al., 1991). This study on a cohort of 75 parents with MS, showed that, among fathers with MS, 21 of 22 had a daughter with MS and only one father had a son with MS. Whereas, among the mothers with MS, 40 had daughters with MS and 13 had sons with MS. This strongly suggests that MS, which has autoimmune aspects, is linked to the X chromosome since the fathers contribute an X chromosome to their daughters but not to their sons. Among sufferers of MS, men more often transmit the disease to their children than women, a phenomenon known as the Carter effect (Kantarci et al., 2006). A role for the X chromosome in this transmission is highly suspected.

We can conclude from the preceding discussion of specific X-linked genes associated with autoimmune diseases that locations all along the X chromosome can potentially be involved in autoimmune diseases (**Figure 2**). However, the most significant vulnerabilities appear to be from Xp21 through PAR1, in effect, the distal portion of the short arm of the X chromosome. Within this section are: the FRAXB fragile site that can delay replication or sustain breaks; polyamine genes that can impact SAM levels; ‘hot’ L1 genes that have functional reverse transcriptase activity; and an abundance of CpG rich Alu elements. The high content of Alu elements in this region requires extensive amounts of methylation and protein suppressors to avoid their inappropriate expression by RNA pol III and potential reverse transcription by L1 or other reverse transcriptases. In the case of the inactive X chromosome, there would be extensive packaging of the DNA to silence genes and this packaging would contain ample supercoiling stress that can add to the dynamics in chromatin disruption (Brooks, 2014). This suspicion of Xp21 through PAR1 is reinforced by rare case reports of non-autoimmune disorders that exhibit autoimmune disease-like symptoms.

**FIGURE 3:**
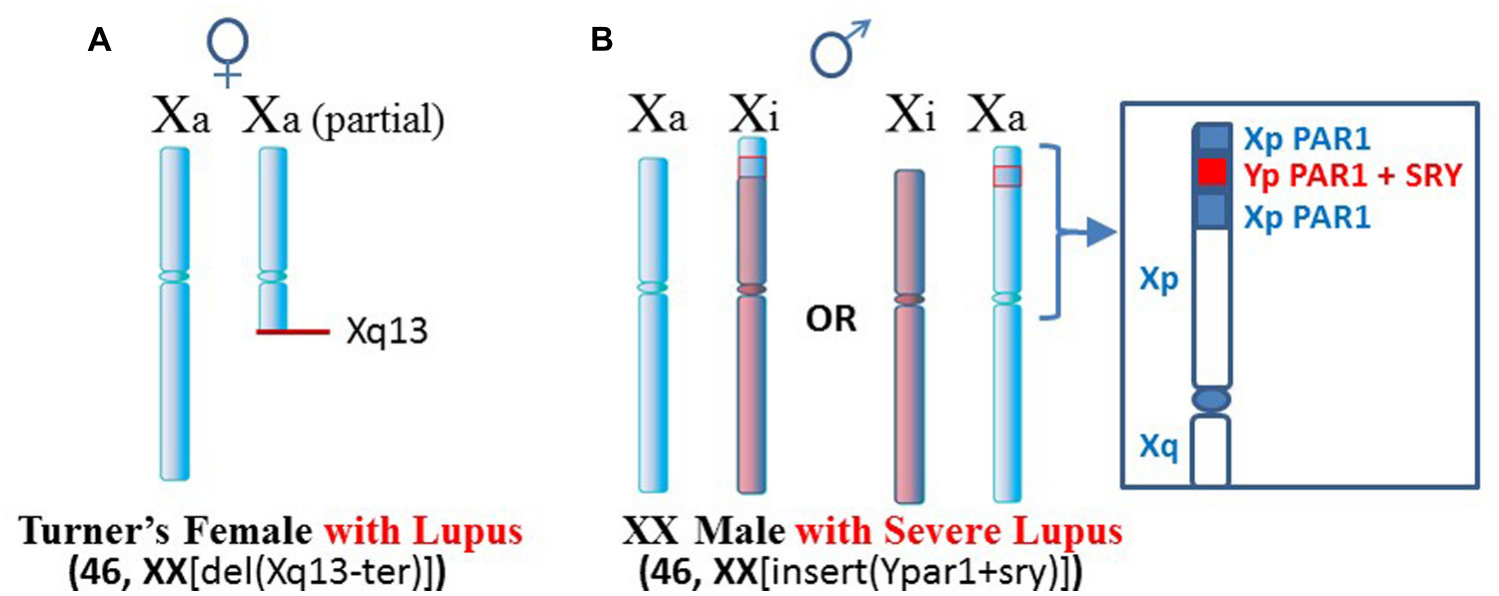
**Lupus in patients with X chromosome abnormalities. (A)** A Turner’s syndrome patient with two X chromosomes but one X is missing distal portions of Xq and does not have a complete XIC at Xq13 (Cooney et al., 2009). As a result, X inactivation cannot occur and there is potential for overexpression from Xp genes. **(B)** An XX male with insertion of portions of the PAR1 and the *SRY* sex-determining gene from the Y chromosome presented severe lupus (Chagnon et al., 2006). Additional chromatin in Xp may be difficult to suppress epigenetically.

One such case, mentioned above, is a Turner’s syndrome patient with a [46, XX del(Xq13-ter)] karyotype who has SLE (**Figure 3A**; Cooney et al., 2009). Establishment of X chromosome inactivation requires an XIC, located at Xq13, in each X chromosome so that early in development, the XICs can be paired and a stoichiometric buildup of interfering RNA transcript expression from the sense and anti-sense strands in the XICs triggers a random choice as to which X, the paternally derived or maternally derived, will be inactivated (Augui et al., 2011). Each daughter cell will then keep that same parentally derived X inactive. However, in this particular patient, only one XIC exists since one X has a deletion of the Xq arm from Xq13 to the terminus. Therefore, proper X inactivation cannot be established. As a result, overexpression of X-linked genes can occur, such as the polyamine genes at Xp22. For example, the *SAT1* gene can undergo superinduction when there is stress from reactive oxygen species (ROS). In this patient’s situation there are two available *SAT1* alleles whereas normally with X inactivation, one would be silenced (Brooks, 2013).

Another case is a male with severe lupus in which the male is actually a [46, XX insert (Ypar1+sry)] karyotype (**Figure 3B**; Chagnon et al., 2006). One X chromosome has insertion in its PAR1 region of the sex determining *SRY* gene from the Y chromosome and a portion of the Y chromosome’s PAR1 region. This gives a male phenotype but there is triplication of some PAR1 genes. X inactivation would still be attempted. In those cells that choose to inactivate the abnormal X, extension of X inactivation into the abnormal region would be difficult due to the low amount of LINE-1 elements for anchoring the XIST RNA. This region could remain active or be easily reactivated. As a result, this patient can have overexpression of X-linked genes in this region.

**FIGURE 4:**
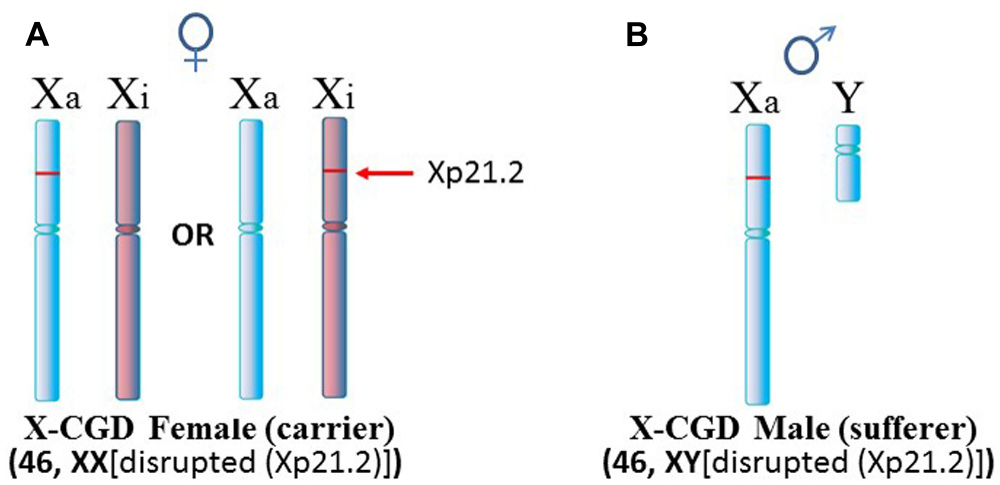
**X-linked chronic granulomatous disease (X-CGD). (A)** X-CGD females are carriers of this recessive disease which is attributed to a variety of abnormalities in the *CYBB* gene at Xp21.2. However, in approximately half the cells, normal *CYBB* would be expressed from the Xa. **(B)** X-CGD males do not have a normal *CYBB* gene and usually succumb to persistent infections at an early age since they cannot clear pathogens properly.

**FIGURE 5:**
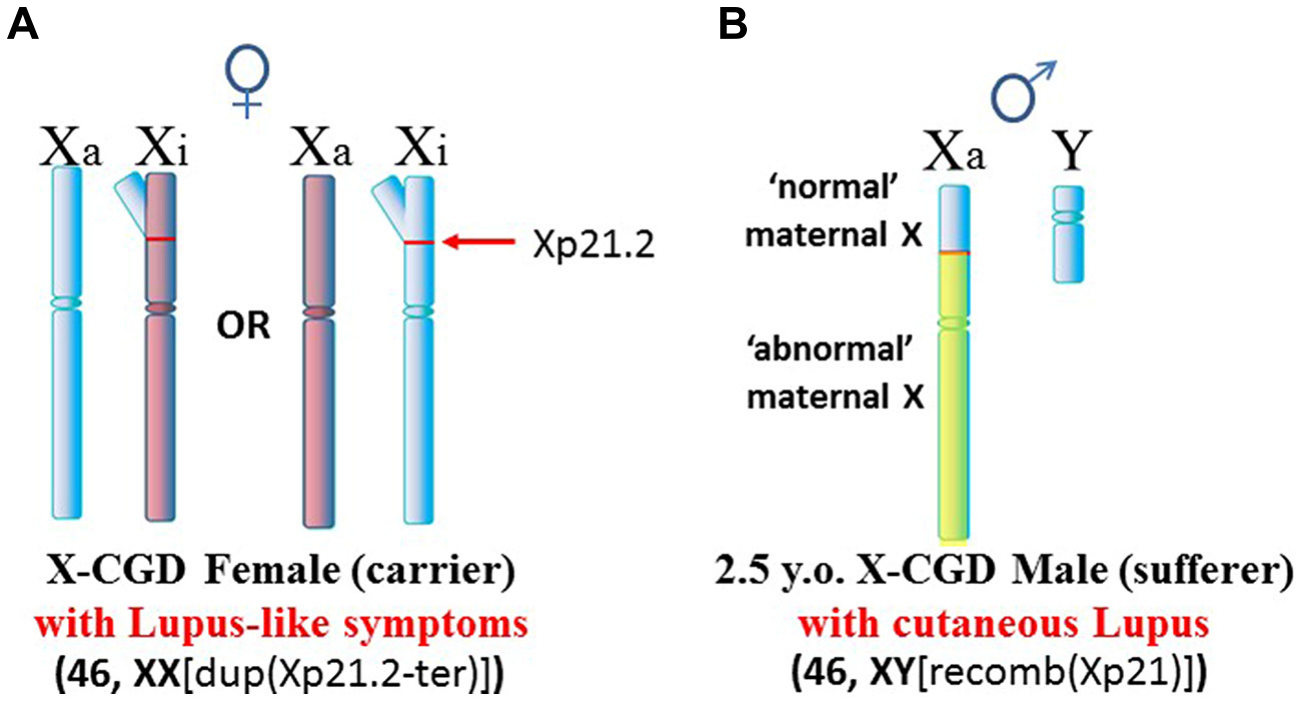
**X-CGD and lupus. (A)** An X-CGD female with duplication of chromatin from Xp21.2 to the distal end of the Xp suffered from lupus-like symptoms (Brandrup et al., 1981). The additional Xp gene copies would present difficulties in maintaining proper X inactivation. **(B)** There are only three reports of lupus-like symptoms in X-CGD males since they succumb at an early age whereas lupus typically appears later in early adulthood. Only one of these cases had data regarding chromosome abnormalities (Ortiz-Romero et al., 1997). In this case, the patient exhibited cutaneous lupus. The X chromosome was a crossover at Xp21.2 between the mother’s normal and abnormal X chromosomes. Whether this X abnormality contributed to the lupus-like symptoms, and how it might contribute, are not clear.

Another situation arises in X-linked chronic granulomatous disease (X-CGD), a rare immunodeficiency disease (**Figure 4**). NADPH oxidase is needed in phagocytes to generate oxygen radicals to destroy phagocytized pathogens. The activated NADPH oxidase consists of several subunits including a cytochrome b component which includes a gp91-phox subunit coded by the *CYBB* gene at Xp21.1 (Roos et al., 1996). The gp91-phox subunit facilitates the interaction of the cytochrome b component with NADPH oxidase. However, in X-CGD, a variety of mutations, insertions, or deletions in the *CYBB* gene disrupt gp91-phox with the end result that the phagocytes cannot clear infections. Mothers are carriers of this recessive disease since they have a second X chromosome with a functional *CYBB* gene. On the other hand, sons are sufferers of X-CGD since they have only the abnormal *CYBB* gene and they usually succumb at an early age to persistent infections that they cannot clear. Although X-CGD normally does not entail autoimmune symptoms, there have been reports in which the *CYBB* gene has insertion of genetic material such that there is duplication of the X chromosome from Xp21.1 to the terminus. Brandrup et al. (1981) reported various lupus-like symptoms in female carriers of X-CGD with the duplication of material from Xp21.1 through the PAR1 (**Figure 5A**). Ortiz-Romero et al. (1997) reported a case of X-CGD with lupus-like lesions in a male sufferer who had a recombinant X chromosome resulting from crossover of the mother’s normal and abnormal X chromosomes (**Figure 5B**). These reports suggest difficulties in the females in establishing and maintaining X-linked dosage compensation. Other reports are available presenting X-CGD with lupus and lupus-like symptoms (Schaller, 1972; Smitt et al., 1990; Manzi et al., 1991; Foti et al., 2004).

**FIGURE 6:**
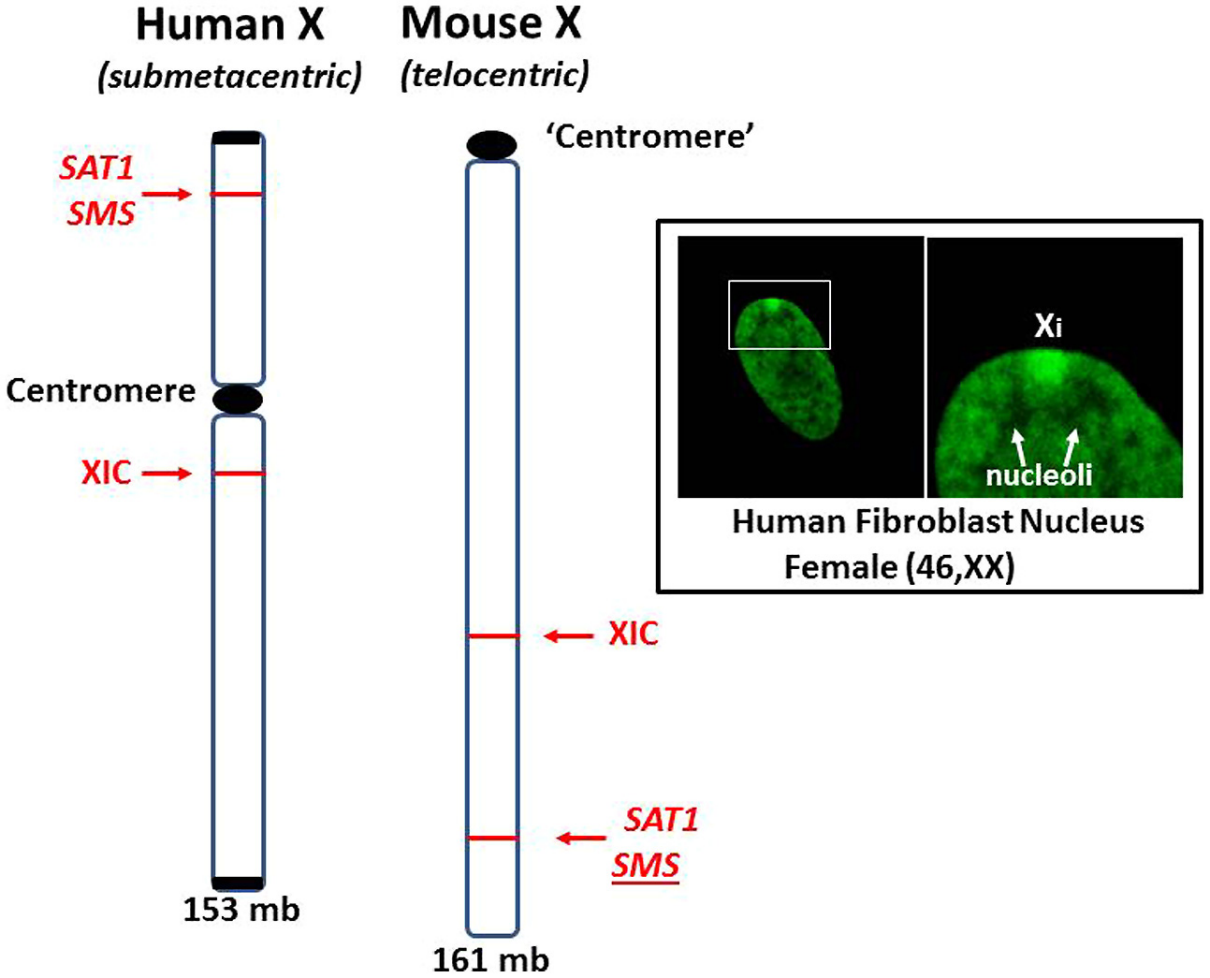
**Comparison of human and mouse X chromosomes.** The human X (153 mb) and the mouse X (161 mb) are ~95% similar in gene content but are significantly different in the arrangement of genes and overall structure (Boyd et al., 2000). The human X is submetacentric with a centromere separating its arms. The mouse X is telocentric with only one arm and a centromere-like structure at one end. X inactivation can spread unencumbered on the mouse X since there is no centromere or L1 gradient to negotiate. For example, the *SAT1* and *SMS* genes are on the other side of the centromere from the XIC in the human X but no such barrier exists in the mouse X. Inset: Human fibroblast stained with fluorescein-tagged anti-histone H1.2 antibodies highlights the inactive X chromosome (Xi), showing its dense heterochromatic character, perinuclear location and its proximity to nucleoli. This places one of the most inactive structures, Xi, close to one of the most dynamic and multi-functional structures, the nucleolus.

These cases of X chromosome abnormalities and autoimmune diseases, along with the female predominance of autoimmune diseases and the increased occurrence of lupus in Klinefelter’s syndrome (47, XXY) males compared to (46, XY) males, strongly suggest that the X chromosome is involved. Furthermore, cases of missing XICs and/or duplication of genetic material of the Xp, particularly from Xp21 to the terminus, suggest the X short arm, Xp, has a key role in autoimmune diseases. Unraveling the contributions of the X chromosome in autoimmune diseases is complicated by the natural phenomenon of X inactivation to achieve dosage compensation of X-linked genes. Studying X inactivation and loss of dosage compensation (a.k.a. reactivation or escape from X inactivation) has proven difficult. Much of our studies on autoimmune diseases have used mice as subjects. Genetically, the mouse has been a good model since the human X (153 × 10^6^ bps) and the mouse X (161 × 10^6^ bps) have 95% of their genes in common (Boyd et al., 2000). However, studies on the epigenetic control of the X chromosome are somewhat problematic due to differing X chromosome structures in the mouse compared to the human (**Figure 6**). The human X chromosome is submetacentric, meaning that there is a centromere between the short arm (Xp) and the long arm (Xq). The mouse X chromosome is telocentric, meaning that there is just one long arm with a centromere at one end (Brown and Greally, 2003). The X inactivation process initiating from the mouse XIC can spread easily throughout the length of the mouse X. In the human X, the X inactivation process must cross the centromere before it can cover the Xp arm and, as mentioned previously, the amount of L1 elements drops in the Xp, providing fewer anchoring sites for the XIST RNA (Ross et al., 2005; Lyon, 2006). Therefore, we would expect genes on the human Xp, including genes such as *SAT1* and *SMS*, to be more vulnerable to reactivation following cellular stresses when compared to genes on the human Xq or on the mouse X. However, this vulnerability is difficult to study since the extent of X inactivation can vary from cell to cell among somatic cells. In addition, when studying the potential for stepwise reactivation of X-linked genes on the human Xi, such as Xp genes, the mouse Xi does not work well as a model since it shows an ‘all or nothing’ response to demethylating agents, either there is no partial reactivation or, with a little more agent, everything on the mouse Xi reactivates. The mouse Xi lacks the distinct differences that exist between the human Xp versus Xq. Another difference between the human and mouse X chromosomes is the content of Alu elements. We have mentioned that Alu elements comprise more than 10% of the human genome and the PAR1 region of the X chromosome is 29% Alu. This high concentration of Alu elements could be problematic if there was sudden exposure to RNA pol III during an Alu stress response as described by Kim et al. (2001). The results could potentially include: disruption of SRP assembly and disruption of extracellular protein synthesis; reverse transcription of Alu elements by L1 or HERV reverse transcriptases; and opening of neighboring genes that were previously sequestered for dosage compensation. On the other hand, the mouse genome does not include any significant amounts of Alu elements but it does have an abundance of B1 elements, which are also short interspersed elements (SINEs) also derived from the 7SL RNA of the SRP, accounting for 7% of the mouse genome (Tsirigos and Rigoutsos, 2009). The mouse genome also contains 0.7% B2 elements which are SINEs derived from tRNA (Ferrigno et al., 2001). Stress can induce increased expression of mouse B1 and B2 SINEs similar to the human Alu stress response (Li et al., 1999). However, it is the high concentration of Alu elements in the human Xp and the location of the inactive X chromosome that could contribute to significant initiating events in human autoimmune diseases.

## PROXIMITY OF THE INACTIVE X CHROMOSOME TO THE NUCLEOLUS

The inactive X chromosome (Xi) is typically observed as a dense heterochromatic structure in a perinuclear location, as if it has been pushed aside by the more active chromatin. This can help explain the late replication of the Xi relative to other chromosomes since it is less accessible but requires more effort in unpacking, repairing, and replicating DNA, and then repacking into heterochromatin. It places greater demands on methylation for DNA, histones, and translocation of other chromatin proteins, such as SAF-A. This is further complicated by the fragile sites with alternate DNA conformations that must be resolved but the transient release of stored negative supercoiling stress from nucleosomes during replication can add to the formation of alternate DNA conformations, such as Z-DNA (Brooks, 2013).

Studies have shown that the Xi associates with nucleoli during S and G2 phases and an estimated one-third of inactive X chromosomes remain in close proximity to the nucleoli during most of the cell cycle (Bourgeois et al., 1985; Zhang et al., 2007). Zhang et al. (2007) proposed that this Xi-nucleolus association may be required to maintain the X inactivation status of the Xi. This spatial association of the Xi and nucleolus then puts the many vulnerabilities of the inactive X chromosome within close proximity of one of the most dynamic and multi-functional components of the cell, the nucleolus (**Figure 6**, Inset). We can begin to imagine the consequences of this Xi-nucleolus association that might occur if a nucleolus increases its synthetic activities to produce ribosomes, tRNAs, SRPs, splicing components, and other macromolecular structures. The nucleolus increases in size, pushing aside most DNA in order to provide space for the folding of nascent RNA transcripts followed by their association with nascent proteins into ribonucleoprotein complexes. As the nucleolus increases in size, it could engulf part or all of the Xi. Since the nucleolar synthesis uses RNA polymerases I and III (RNA pol I and III) to produce many of the specialized non-coding RNA transcripts, the Xi could then be exposed to RNA pol III that could transcribe many of the Alu elements, including in PAR1. RNA pol III transcribes genes for non-coding RNAs such as the 7SL RNA of the SRP, the U6 RNA, the 5S rRNA of the ribosome, tRNAs, and SINEs (MIR and Alu; Noma and Kamakaka, 2010). The nucleolus contains abundant RNA pol III including a perinucleolar compartment that contains a very high concentration of RNA pol III protein and nascent transcripts (Matera et al., 1995). Many of these transcripts are associated with the Ro and La proteins that are frequently autoantigens in SLE and are believed to serve as chaperones in the early processing of RNA pol III transcripts (Rinke and Steitz, 1985). We should also note that RNA pol III requires fewer transcription factors than RNA pol II and does not require ATP to support its progression.

Now consider the Xi-nucleolus interactions when there is cellular stress that results in an Alu stress response and/or a nucleolar stress response. We should focus, in particular, on the cluster of Alu elements in the X PAR1 region of the Xp and its close proximity to RNA pol III in the nucleolus. Other Xi vulnerabilities in this region are also important to consider: the ‘hot’ L1 elements, the fragile sites that can delay replication and the polyamine genes, *SAT1* and *SMS*, which compete for SAM, the methyl donor.

The Alu stress response can lead to selective opening and expression of Alu transcripts (Liu et al., 1995; Kim et al., 2001). The Alu stress response involves shifting of nucleosomes to expose the intragenic RNA pol III promoter sites and displacement of any Alu binding proteins. Two proteins that have been proposed as Alu binding proteins are the Alu co-repressor 1 (ACR1) protein (Kropotov et al., 1999) and the Ku antigen which binds a GGAGGC motif in the Alu core sequence, possibly in association with the TATA-binding protein (TBP; Tsuchiya et al., 1998). Opening of the Alu elements would release stored negative supercoiling stress from the disrupted nucleosomes and that stress will flux through the region transiently disrupting other nucleoprotein complexes, such as those involved in the XIST RNA anchoring or the supercoiling stress could transiently flip stretches of DNA into alternate conformations, such as Z-DNA, which could be stabilized by increased polyamines and nuclear aggregates of polyamines (NAPs; Brooks, 2013).

Nucleolar stress responses can take many forms and involve changes in the nucleolar morphology (Hernandez-Verdun et al., 2010). Often it entails redistribution of nucleolar proteins, changes in the active synthetic pathways being processed and even changes in the size of the nucleolus. Boulon et al. (2010) refer to the “spatial proteomics” of the nucleolar proteins since the changes can include redistribution between the nucleoli, nucleus, and cytoplasm. As for the size changes, depending on the type of stress, the nucleolus may appear to shrink and even dissolve but viral infections tend to cause an increase in nucleolar size as the active virus induces the nucleolus to increase production of ribosomes and tRNA for viral protein synthesis and increase expression and processing of viral RNA transcripts (Hiscox, 2007; Greco, 2009).

These stress induced changes in the nucleolus could potentially impact the nearby Xi, even engulfing the Xi and exposing it to nucleolar contents. In this scenario the abundant Alu elements in Xp PAR1 could be exposed to the high levels of RNA pol III in the nucleolus. This could lead to a sudden increase in Alu transcripts in the nucleolus that could interfere with the SRP assembly and function, as described previously (Brooks, 2012). Briefly, competition between Alu domains in the 7SL RNA of the SRP and the Alu RNA transcripts from Xp PAR1 and elsewhere would lead to incomplete SRPs that cannot halt translation by the ribosome when the signal recognition domain reads the signal for an extracellular protein. In such a scenario, the extracellular protein would be expressed in the cytoplasm rather than the endoplasmic reticulum and the nascent protein would be inappropriately exposed to cytoplasmic enzymes, such as peptidylarginine deiminases (PADs) and transglutaminases. The abundance of Alu RNA transcripts could be reverse transcribed by ‘hot’ L1 reverse transcriptases, such as the ones at Xp22, which could create an abundance of hypomethylated Alu DNA (Brooks, 2002). This could explain the abundance of Alu DNA (55% of the free DNA) in SLE sera as reported by Li and Steinman (1989).

## POLYAMINE INTERACTIONS IN THE NUCLEOLUS

**FIGURE 7:**
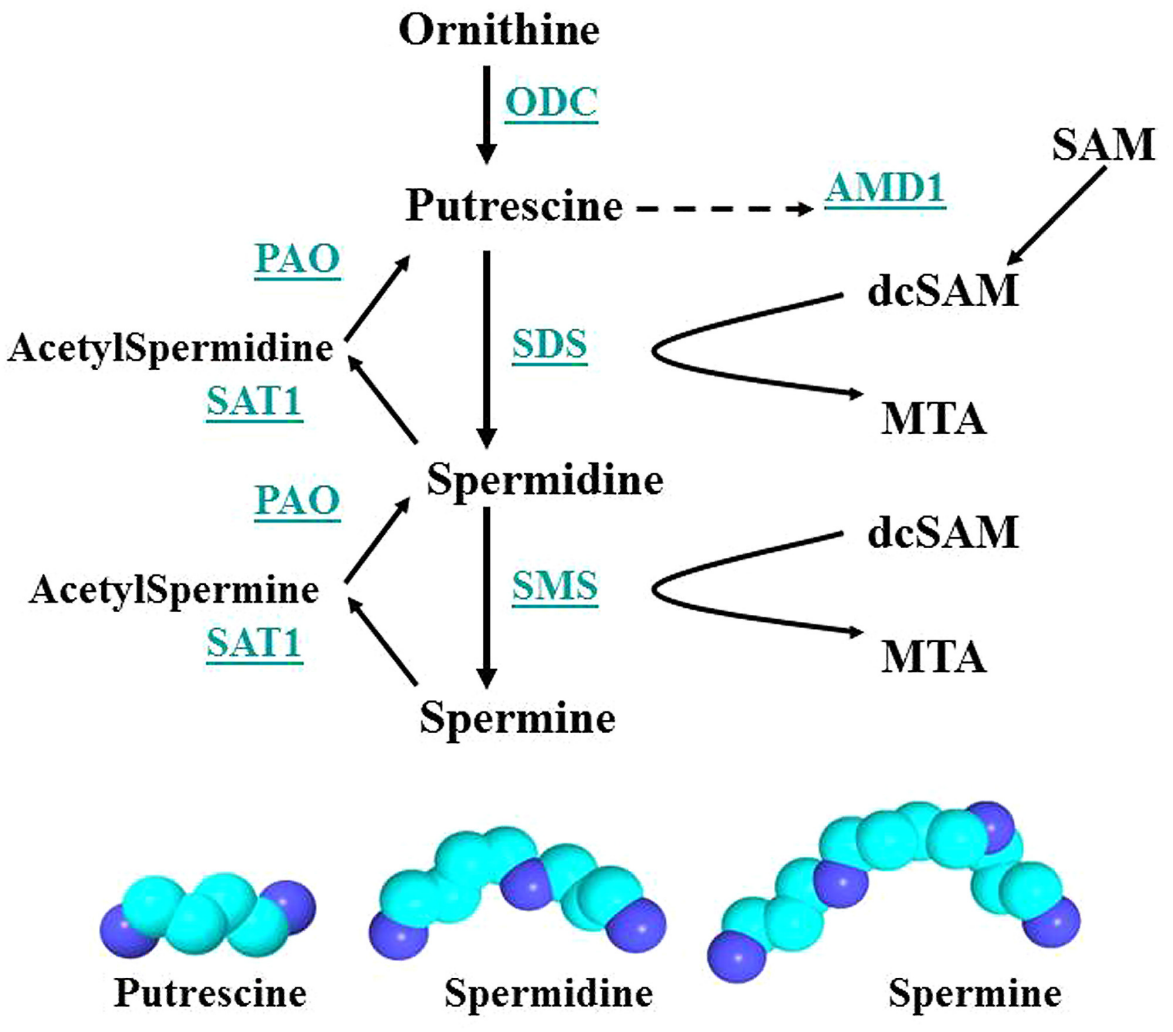
**Polyamine synthesis and recycling.** Polyamine synthesis is tightly controlled since it competes for SAM with cellular methylation (DNA and histone methylation, protein and RNA localization facilitated by methylation). ODC and AMD1 are key enzymes since ODC produces putrescine, the polyamine precursor, and putrescine can allosterically increase AMD1 activity. However, induction of SAT1 can recycle polyamines to putrescine, which then activates AMD1 and polyamine synthesis. Key: ODC, ornithine decarboxylase; SAM, *S*-adenosylmethionine; PAO, polyamine oxidase; AMD1, SAM decarboxylase; SAT1, spermidine/spermine-*N*1-acetyltransferase; dcSAM, decarboxylated SAM; SDS, spermidine synthase; MTA, methyl thioadenosine; SMS, spermine synthase.

The polyamines are highly charged polycations that serve many essential functions in the cell (**Figure 7**; Moinard et al., 2005; Pegg, 2009; Igarashi and Kashiwagi, 2010). For example, polyamines can control splicing and translation of their own enzymes for synthesis (ornithine decarboxylase, ODC; Persson et al., 1988) and recycling (spermidine/spermine *N*1-acetyltransferase; Hyvönen et al., 2006). Polyamines are important for modulating changes in chromatin structure (Visvanathan et al., 2013) and they are important for regulating RNA synthesis in the nucleolus (Whelly, 1991). For this discussion, we will focus primarily on the ability of polyamines to stabilize alternate conformations of nucleic acids and on the ability of polyamines to aid in RNA folding, protein folding and nucleoprotein complex assembly that occurs in the nucleolus. In fact, the majority of spermidine and spermine are believed to be associated with RNA and, to a lesser extent with DNA. In addition, the nucleolus, site of RNA folding and ribonucleoprotein assembly (SRP, ribosomes, tRNAs, splicing components, and more), has a high concentration of polyamines that can change rapidly as needed in response to cell cycling and cellular stresses (Gfeller et al., 1972; Shin et al., 2008). We should still bear in mind, however, the impact of increased polyamine synthesis which lowers the levels of SAM available for cellular methylation important in epigenetic silencing and protein and RNA trafficking.

RNA folding is more efficient with higher charged counterions. Divalent ions like Mg^+2^ require millimolar concentrations whereas trivalent ions like spermidine can work at micromolar concentrations in *in vitro* experiments that follow steps in RNA folding (Koculi et al., 2004; Woodson, 2004). The dense charge of a Mg^+2^ ion can organize multiple water molecules into a shell of one or more hydration layers around the Mg^+2^ making it a bulkier hydrated counterion when it does interact with RNA (Draper et al., 2005). And so the charge density and distribution over the length of the counterion is a factor. The +3 charge of spermidine is spread over ~13 Å with the individual +1 charged amines separated by alkyl linkers, thereby reducing the extent of organized hydration. On the other hand, the +3 charge of a hexamminecobalt(III) counterion is focused at the metal but has a 6 Å length due to the amino groups. The hydration shell is more compressible such that, in experiments with DNA, water molecules can be displaced and at least two direct interactions of the hexamminecobalt counterion with the nucleic acid can be formed (Kankia et al., 2011). As such, with the combination of dense high charge and compressible hydration, the hexamminecobalt is more efficient at *in vitro* experimental RNA folding than spermidine (12 μM cobalt hexamine vs. 55 μM spermidine) but the spermidine is the more relevant counterion for the *in vivo* setting (Woodson, 2004). The polyamines, spermidine and spermine, provide a very effective combination of charge and length for the dynamic hydration and dehydration of nucleic acids and counterions that are important in facilitating RNA folding and the polyamines are effective at lower concentrations than competing counterions. The nucleolus is the site of much of the cell’s RNA folding activity and, therefore, we see a close relation between polyamine levels and nucleolar activity.

**FIGURE 8:**
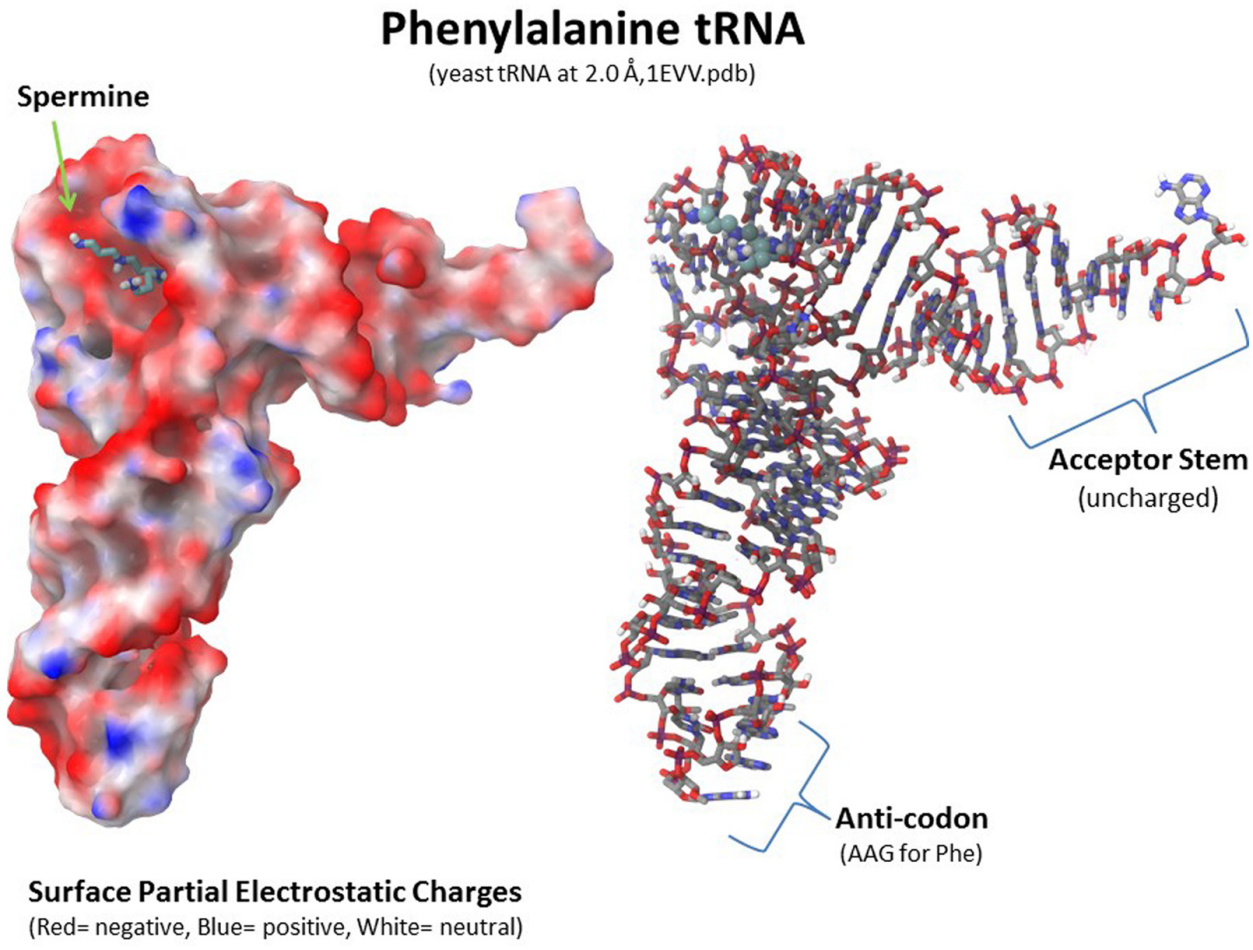
**Polyamine involvement in RNA folding and ribonucleoprotein assembly.** Spermine is involved at a key location in tRNA to stabilize the final RNA conformation as seen in the yeast phenylalanine tRNA structure (1EVV.pdb from the Protein Data Bank: www.rcsb.org; Jovine et al., 2000). Polyamines are involved in RNA folding and ribonucleoprotein assembly in nucleoli as transient factors initiating folding and as stabilizing factors in the final macromolecular complexes. Xaplanteri et al. (2005) identified more than 30 sites in the 23S rRNA and more than 100 sites in the 50S ribosomal subunit at which spermine or spermidine could potentially interact with the RNA and proteins.

Polyamines are important for RNA folding but they are not necessarily a component of the final RNA or ribonucleoprotein moiety. As shown in **Figure 8**, a spermine molecule is located in the final structure at a central site in the yeast phenylalanine tRNA where the spermine can stabilize the bent RNA structure (Jovine et al., 2000). On the other hand, a yeast branchpoint-U2 snRNA structure does not show any spermine molecules in the crystal structure but spermine at 1–3 mM is required during the crystallization to obtain the structure including the unpaired adenosine bases that protrude out as part of their function in the nucleophile attack on 5′ splice sites (Berglund et al., 2001). In this structure, the spermine may be randomly positioned and not provide sufficient diffraction data to determine the spermine’s position(s) or the spermine is required only transiently in the folding process. Another interesting study investigated binding sites for spermine or spermidine in the 23S rRNA and the 50S subunit of the bacterial ribosome, which consists of the 23S rRNA, a 5S rRNA, and 33 proteins (Xaplanteri et al., 2005). Using a photoactivated crosslinking agent, *N*1-azidobenzamidino spermine (ABA-spermine), the authors identified more than 40 sites in the 23S rRNA and more than 135 sites in the 50S subunit where the polyamines could potentially interact with the RNA. This suggests that the polyamines could help with the initial folding of the 2,094 bases of the 23S rRNA and be involved in the final ribonucleoprotein assembly.

Although the polyamines are important in RNA folding and assembly of macromolecular complexes and the polyamine levels increase during stress to facilitate increased production of ribosomes, tRNAs, SRPs, and splicing components, there can be consequences if the polyamine levels are not controlled. An increase in putrescine can support the formation of NAPs which can stabilize infrequent conformations in DNA, RNA, and proteins (Brooks, 2013). At the same time, the larger NAPs would not be as efficient as individual spermine or spermidine molecules in RNA folding. Another potential problem with excess polyamines in the cell is that polyamine recycling can generate acrolein. Acrolein is very reactive and can be toxic to cells. Appearance of acrolein conjugated proteins closely parallels the severity of SjS episodes (Higashi et al., 2009). In addition, polyamines can be conjugated to proteins by transglutaminases, potentially altering their immunogenicity (Haddox and Russell, 1981; Agostinelli, 2014). And we should not overlook the allosteric effect of putrescine on AMD1 in reducing SAM levels, thereby impacting cellular methylation required for DNA and histone epigenetic control and for RNA and protein movement among cellular locations. Finally, the polyamines can impact the rate of Xi replication by stabilizing alternate DNA conformations, such as in fragile sites. This can slow the late S phase replication and reformation of Xi heterochromatin significantly so that portions of the Xi may not complete their replication until after S phase, if they complete at all. The Xi then becomes vulnerable to altered gene expression, mutations, breaks, translocations and even loss of the Xi. It has been reported in the context of breast cancer that loss of the Xi can lead to duplication of the active X which would result in overexpression of X-linked genes (Richardson et al., 2002). Such duplication has been reported in autoimmune diseases (Invernizzi et al., 2009). And we should suspect that, on some occasions when there is difficulty with the Xi, such as delayed replication, the subsequent segregation of chromosomes could be affected such that there is uneven distribution between daughter cells resulting in a mosaic of XO, XX, and XXX cells.

## GENERATION OF AUTOANTIGENS

When a pathogen enters a cell and uses the cellular machinery to replicate itself, the nucleolus is a prime target for the pathogen because of the importance of the nucleolus in cellular synthesis and replication (Boisvert et al., 2007; Bierne, 2013). The pathogen requires the cell’s ribosomes and tRNAs to synthesize the pathogen’s proteins. And the pathogen can use the cell’s polymerases to generate pathogen RNA transcripts which are folded in the nucleolus. Many pathogens will first induce increased polyamine synthesis to support the increased nucleolar activity they require (Goyns, 1981). We use Epstein-Barr virus (EBV) as a prime exemplary pathogen to convey the concepts leading to tissue degeneration and autoimmune responses attributable to pathogen infections. Other pathogens, such as hepatitis B virus (HBV; Maya et al., 2008) and varicella zoster virus (VZV; Rodriguez-Violante et al., 2009), are also proposed as having a role in some cases of autoimmune diseases, and those pathogens may follow similar patterns as EBV in the development of autoimmune diseases. In fact, it may be that, once the host cells are compromised by one pathogen, subsequent stress by other pathogens (bacterial or viral) or agents (heavy metals, heat shock, or drugs) can follow similar paths and trigger additional bouts that take advantage of the prior disruption. In the case of EBV, it induces increased c-MYC activity (Bajaj et al., 2008) which induces increased expression of *ODC*, *SMS* and *SDS*, thereby increasing polyamine synthesis and consumption of SAM (Bello-Fernandez et al., 1993; Dang, 1999; Nilsson et al., 2005). EBV also upregulates RNA pol III transcription to generate RNA for ribosomes and tRNAs as well as for expression of viral RNAs ( Gomez-Roman et al., 2003; Felton-Edkins et al., 2006). This could entail active transcription from newly exposed Alu elements.

**FIGURE 9:**
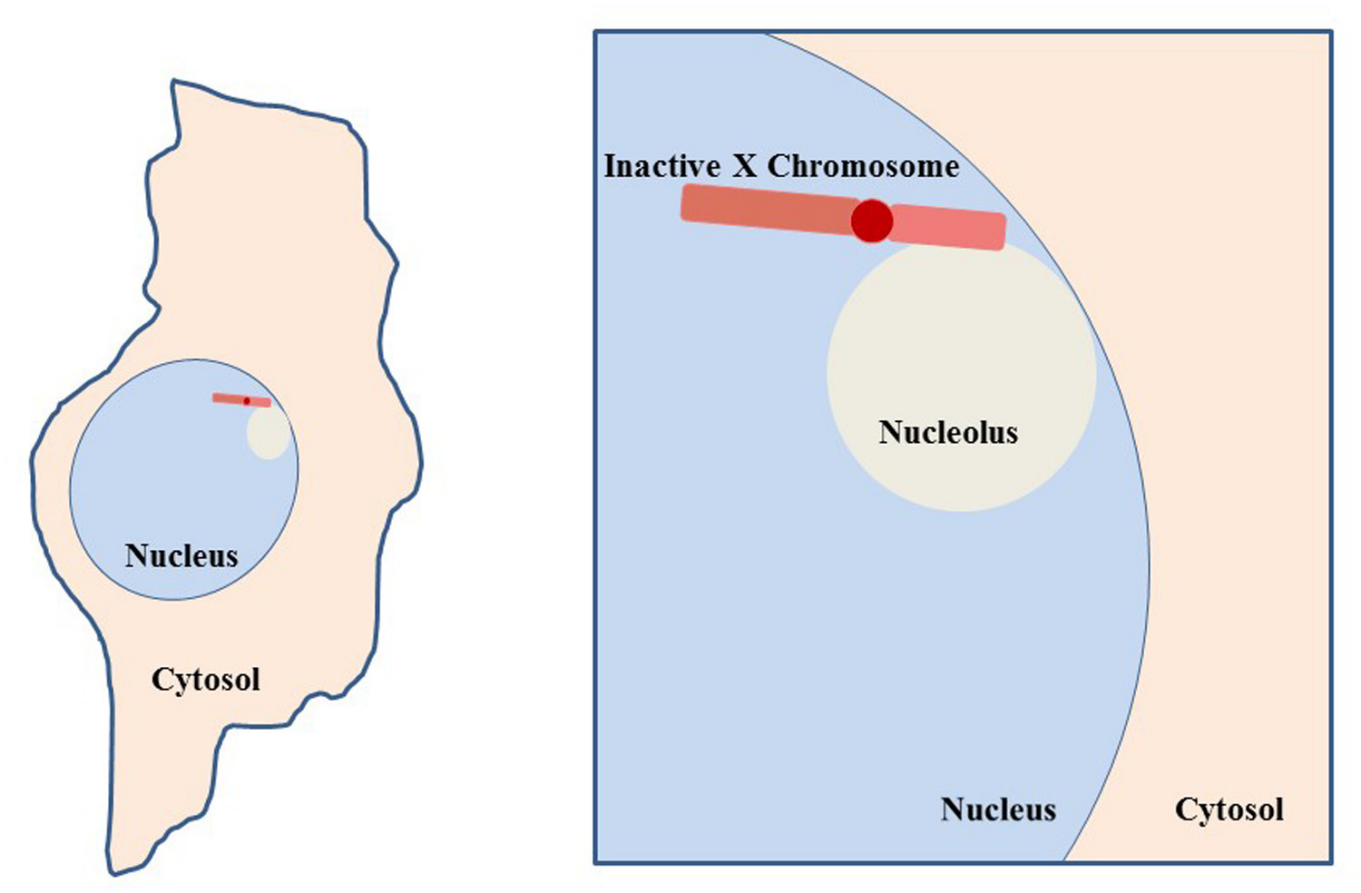
**Co-localization of inactive X chromosome with the nucleolus.** Depiction of a typical cell, such as a fibroblast, with the nucleolus and Xi in close proximity. This association suggests involvement of the nucleolar machinery in maintenance of the inactive state of the Xi. The Xi is seen in this close association 80–90% of S phase cells and approximately one-third of cells throughout interphase (Zhang et al., 2007).

**FIGURE 10:**
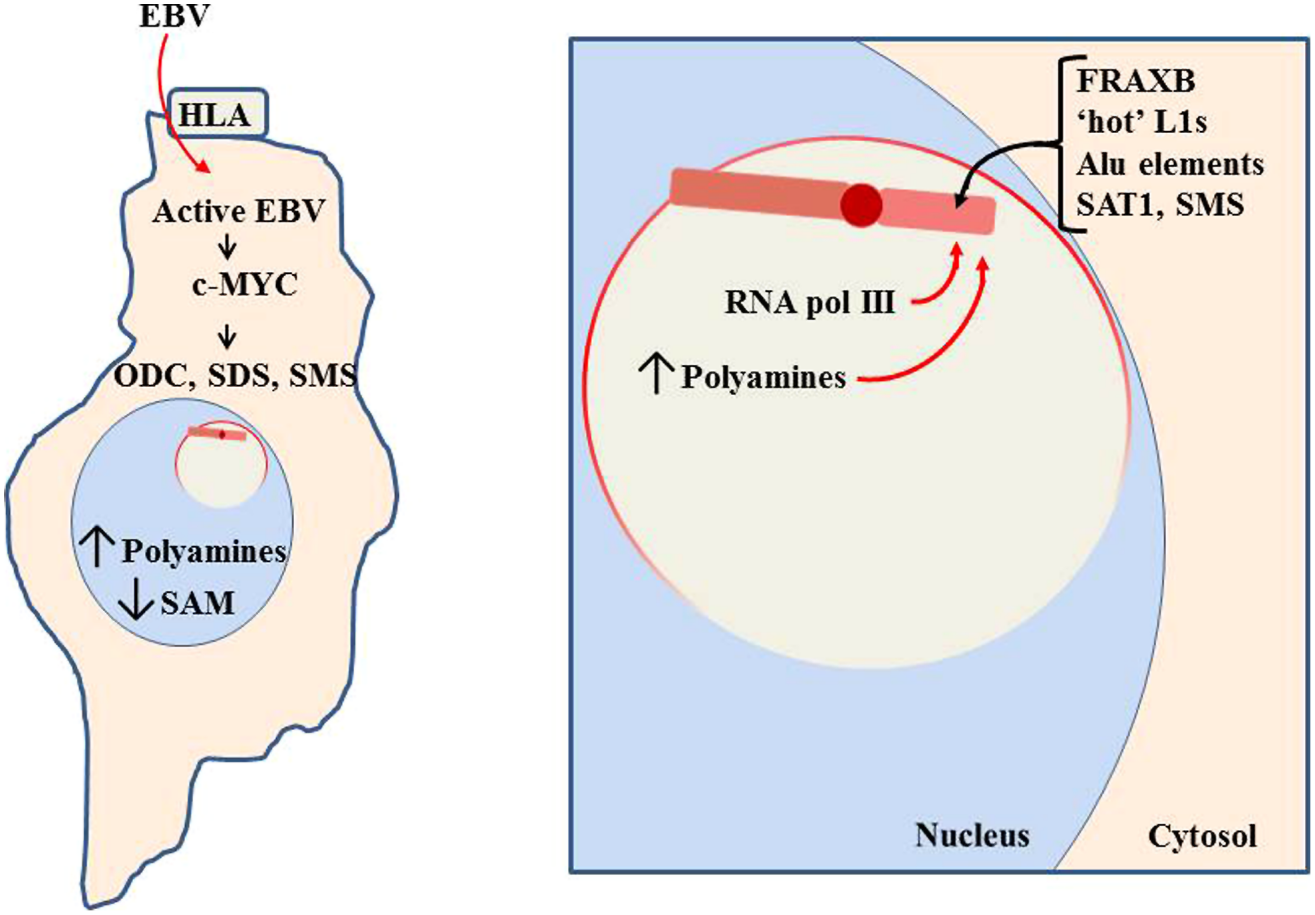
**Pathogen impact on nucleolar activity.** When a pathogen, such as Epstein-Barr virus (EBV), enters a cell, it will attempt to use cellular machinery to replicate itself. This includes using the nucleoli to process pathogen RNA transcripts and increase ribosomes and tRNAs to produce the pathogen’s proteins. In the case of EBV, it can use the human lymphocytic antigen (HLA) to enter the cell (Li et al., 1997). It then induces increased RNA pol III and c-MYC activity, which induces increased activity of polyamine synthesis enzymes: ODC, SDS, and SMS. This reduces SAM while increasing polyamines. Much of the polyamines localize to the nucleoli to aid in RNA processing, thereby causing the nucleoli to swell in size as more room is needed for the increased processing. The enlarged nucleoli could potentially engulf the Xi and expose the Xi to the nucleolar abundance of polyamines and RNA pol III.

**FIGURE 11:**
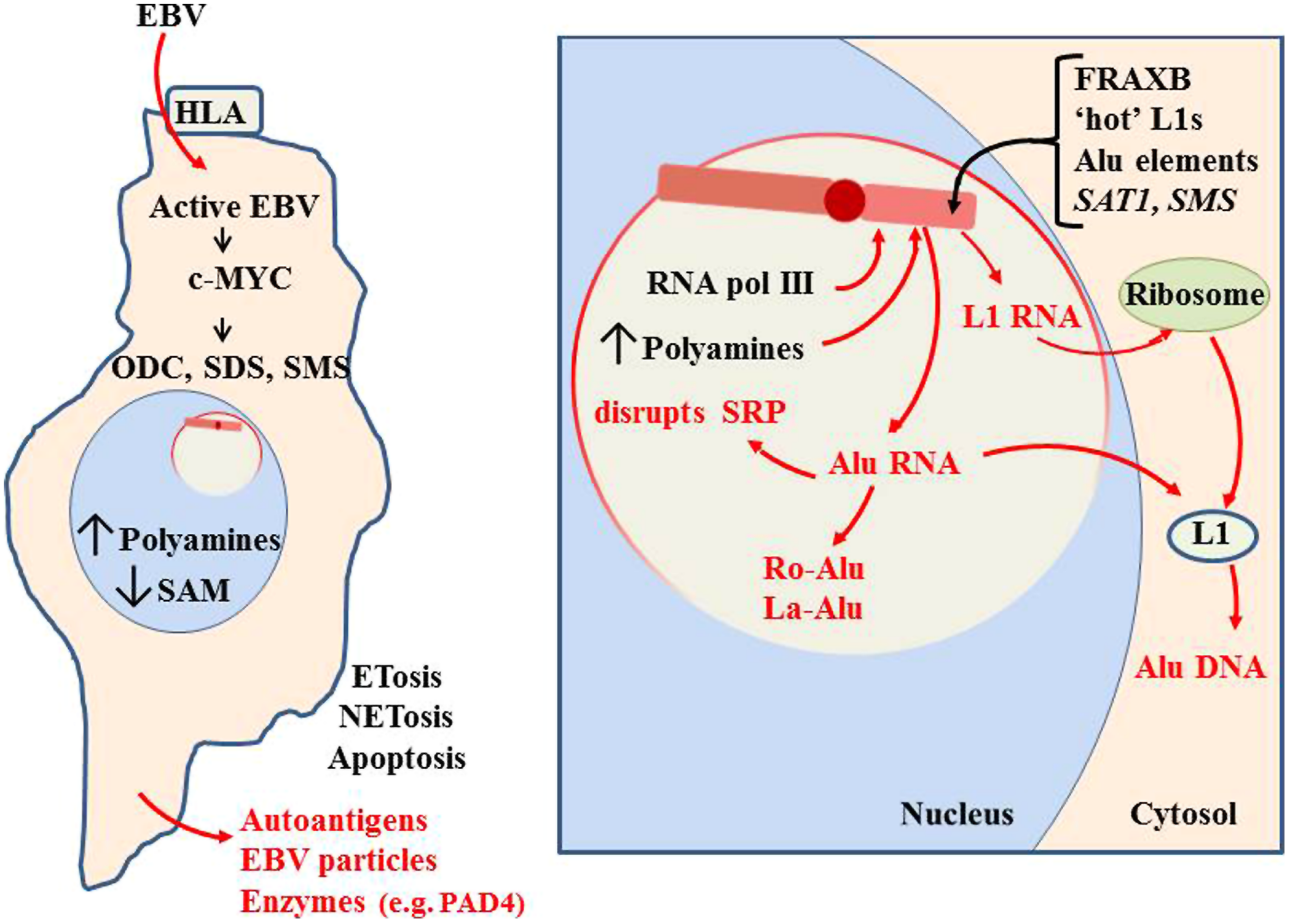
**Initiation of autoantigen formation.** Polyamines and nuclear aggregates of polyamines (NAPs; Brooks, 2013) could stabilize alternate DNA conformations in the Xi which could slow replication and packaging of Xi chromatin, especially in fragile sites. Disruption of the Xi could expose Alu elements to RNA pol III, generating an abundance of Alu RNA. Excess Alu RNA can disrupt SRP assembly leading to difficulties in translation of extracellular proteins (Brooks, 2012). Alu RNA may be stabilized in autoantigenic conformations by polyamines or NAPs. Association of Alu RNA with Ro and La proteins, which aid in processing of RNA pol III transcripts, could incorporate Ro and La into autoantigens. Expression of L1 from the Xi could lead to L1 protein that reverse transcribes Alu RNA to DNA. This Alu DNA would be hypomethylated and could bind Ku and nascent histones in the cytosol. These would be autoantigenic since they are outside their normal epigenetic context. These autoantigens could be released extracellularly through apoptosis, NETosis or ETosis. This can include degradative enzymes, such as peptidylarginine deiminase 4 (PAD4; Brooks, 2013).

As the nucleolus swells with the abnormal increases in polyamines and RNA processing, the nucleolus can engulf the Xi and expose it to the polyamines and RNA pol III (**Figures 9** and **10**). With the increased polyamines and RNA transcripts, including Alu RNA, there can be misfolding of RNA and potential stabilization of this abnormal RNA by polyamines and NAPs. For example, Alu RNA can form cruciforms that could be stabilized by polyamines and NAPs into autoantigens. These RNA transcripts can incorporate the Ro protein into autoantigenic complexes since the function of Ro is to identify misfolded RNA (**Figure 11**; Stein et al., 2005). Ro is often an autoantigen in lupus. Excess polyamines and NAPs may hinder refolding or degradation of the RNA leaving the complex as an autoantigen. Likewise, The La protein associates with RNA pol III genes to chaperone the transcripts through nucleolar processing (**Figure 11**; Fairley et al., 2005). In an autoantigenic complex of polyamines, RNA and Ro or La, the proteins (Ro and La) would be constant epitopes and thereby take part in eliciting an autoimmune response (anti-SSA/Ro, anti-SSB/La antibodies). Epitope spreading, initiated from autoantigenic RNA, DNA or proteins could eventually involve reactions to less conformationally compromised material, i.e., the more natural endogenous conformations could be targeted in later stages of an autoimmune response.

The excess Alu RNA transcripts generated from the Xi can disrupt the assembly of SRP since the Alu RNA can compete with the Alu domain in the 7SL RNA of the SRP for SRP proteins SRP9 and SRP14 (Brooks, 2012). This can leave the SRP lacking the ability to halt translation of extracellular proteins by the ribosome, exposing those proteins to intracellular enzymes they would not normally encounter. In addition, the excess Alu from the Xi could become substrate for reverse transcription by L1 reverse transcriptase protein that may be expressed from the Xi. The reverse transcribed Alu DNA would be hypomethylated if it is created in the cytosol away from the nuclear DNMTs. Also, these transcripts could bind the Ku protein, another frequent autoantigen in lupus, since Ku binds GGAGGC sites found in Alu DNA (Tsuchiya et al., 1998; Tutrjs and Tuteja, 2000). This rapid generation of Alu DNA could also yield more Alu RNA since the Alu elements have an internal RNA pol III site. The abundance of Alu DNA that would result from reverse transcription can help explain the observation by Li and Steinman (1989) that free DNA in lupus patients’ sera is 55% Alu and the observation of Alu retrotransposition induced by stress (Hagan et al., 2003). Nucleolin, a major protein in the nucleolus, is involved in decondensation of chromatin, processing of RNA transcripts and maturation of ribosomes. Nucleolin can be targeted as an autoantigen (Minota et al., 1991). This may also be due to the abnormal activity occurring during nucleolar stress. Other nucleolar material, such as tRNAs and splicing components, could be stabilized in abnormal conformations or complexes with polyamines and due to imbalance in RNA processing components, such as Ro and La.

One final concept that we should consider is that the increased polyamines and NAPs in the nucleolus and nucleus could disrupt miRNA and lncRNA conformations and processing such that there is dysregulation of the expression levels of their targeted genes. The broad multi-target aspects of miRNAs could result in quite expansive disruption of protein expression in cells with very complicated effects.

## FUTURE DIRECTIONS

Much of the research on autoimmune diseases in the past has taken a genetic approach with the idea that we would find the elusive ‘lupus’ gene or the ‘MS’ gene, much like the concept that mutations in a specific gene often have an important role in cancers. SNP studies and genome-wide association studies (GWAS) have identified genes that have statistical significance in specific autoimmune diseases but that still leaves a vague picture of the ‘cause and effect’ in explaining triggering events and progression. Epigenetics as a factor in autoimmune diseases has been gaining interest in the past decade but many researchers still have a genetics-based perspective of epigenetics, that screening for the methylation state of genes will uncover the hypomethylated or hypermethylated ‘lupus’ gene. But epigenetics also involves sections of chromatin between genes such that potential problems can initiate without specific genes being involved. For example, release of DNA supercoiling stress stored in nucleosomes can flux through the chromatin and disrupt sites 1000s of bps away, opening loops of chromatin with genes and pseudogenes that were previously suppressed but now have exposed promoter regions. If we think of Alu elements with all their copies as being genes, albeit small non-coding genes, then there is not one single ‘lupus’ gene but more than a million. That still leaves us short of an explanation for the triggering and progression of autoimmune diseases. We have to think of the higher levels of epigenetics that can be involved, such as X inactivation and its potential for disruption with subsequent overexpression or loss of X-linked genes. And we have to think of epigenetic control as stored potential, not just for individual gene expression, but for DNA stress that can have far reaching impact on gene packaging and DNA conformations. Epigenetic changes can also involve subtle events, such as transient appearance and possible stabilization of alternate DNA conformations in a fragile site that then delay the completion of DNA repair or replication, placing the entire chromosome in jeopardy of degradation or misallocation during cell division so that daughter cells inherit different amounts of chromatin. Among the many components in epigenetic control (small non-coding RNAs, histones, MECP2, counter ions, etc.) the polyamines are particularly interesting due to their versatility, their unique combination of charge and length, and their involvement in RNA folding, nucleoprotein assembly and episodes of cellular stress. In addition, their ability to form aggregates, NAPs, when sufficient putrescine, spermidine, and spermine are available can be potentially even more detrimental by stabilizing autoantigenic conformations of nucleoprotein complexes. The importance of polyamines in nucleolar activities places the polyamines in a critical position during cellular stress when the most inactive component in the nucleus, the inactive X chromosome with all its vulnerabilities, could be engulfed by the most active and dynamic component of the nucleus, the nucleolus. Looking at a list of autoantigen types in lupus, the mystery clears somewhat as we realize that most can be grouped as components of chromatin (DNA, histones) or components of the nucleolus (Ro, La, RNA, etc.). Still, we must keep in mind that there are other possibilities. For example, the DNA may be reverse transcribed DNA in the cytosol and the histones may be nascent histones in the cytosol that bind the reverse transcribed DNA.

The differences between the human and mouse X chromosomes present difficulties in using mouse models of autoimmune diseases. On one hand, the mouse adaptive immune response can give us good insights on events in the human adaptive immune response. On the other hand, X-linked events that precede the adaptive immune response may not compare as well since the human X appears more vulnerable to loss of dosage compensation and has unique problematic components, such as Alu elements, “hot” L1 elements, and X-linked polyamine genes that may reactivate more readily. The mouse X chromosome does not present these same vulnerabilities to the degree that we can model progressive loss of dosage compensation suspected to occur in the human Xi. On the other hand, the mouse models do show the possible involvement of polyamines since spontaneous lupus symptoms in the NZB/W lupus mouse model can be suppressed with the addition of difluoromethylornithine (DFMO) to the drinking water (Thomas and Messner, 1991). DFMO is a known inhibitor of ODC , the initial enzyme in polyamine synthesis. The Jimpy and Quaking mouse models show an accumulation of polyamines that accompanies an increasing myelin deficiency (Russell and Meier, 1975). This could be modeling the tissue damage that occurs in human MS. And overexpression of *SAT1* in mice leads to hair loss (Suppola et al., 1999). This could be a model of our proposed overexpression of human X-linked *SAT1* in autoimmune diseases when there is disruption of the inactive X chromosome with subsequent loss of dosage compensation for *SAT1*. Hair loss occurs as a symptom in lupus and MS.

We expect that, as the autoimmune disorder research community gains more understanding of epigenetics and its potential, there will be more projects that are based on hypotheses of epigenetics involvement and less emphasis on finding specific genes and mutations. In addition, while mouse models are of value in understanding the adaptive immune response, the mouse models must be reappraised for their value in studying the innate immune response and early events that precede the autoimmune reaction. Projects targeting the adaptive immune response are of value in determining how to reduce the autoimmune reaction however our hypotheses suggest that projects that increase our understanding of the innate response, cellular and nucleolar stress responses and the stability of the inactive X chromosome, may well prove to be more beneficial in preventing and curing autoimmune disorders. Drug discovery targeting PAD enzymes to reduce NETosis, targeting pathogens, such as EBV, targeting other enzymes involved in epigenetic control and targeting polyamine synthesis and recycling enzymes should prove of greater value for eventual clinical use for prevention and control of autoimmune disorders.

## SUMMARY

We have described the vulnerabilities of the inactive X chromosome which include its need for extensive heterochromatic assembly and maintenance, its perinuclear localization, its proximity to the nucleolus, and the potential detrimental actions that can arise from Alu elements, L1 elements, fragile sites, polyamine genes, miRNAs, and HERVs. The nucleolar stress response, such as the impact of pathogen activity exemplified by EBV, can lead to engulfment of the Xi by the nucleolus and exposure of the Xi to the high concentrations of polyamines and RNA pol III. Abnormal expression of previously sequestered alleles on the Xi, such as miRNAs and Alu and L1 elements, can alter the nucleolar activity and generate polyamine-stabilized (and possibly NAP-stabilized) alternate (misfolded) conformations of RNA and DNA, proteins and nucleoprotein complexes that are potentially autoantigenic. Constant epitopes, such as Ro, La, or Ku proteins, within these abnormal nucleoprotein complexes could also become autoantigenic targets. These autoantigens can then be exposed extracellularly where they elicit an autoimmune reaction. The extracellular exposure of these autoantigens can occur through apoptosis, NETosis (neutrophil extracellular trap release), ETosis, or cell surface presentation. This hypothesis: ‘X chromosome-nucleolus nexus’ fits well with the previously published ‘NAPs in NETs’ hypothesis (Brooks, 2013). Together these hypotheses provide explanations for many of the autoantigens encountered in autoimmune diseases and they point to loss of epigenetic control, particularly in relation to the Xp arm of the inactive X chromosome, as being of significance in the mechanisms of autoimmune diseases.

## Conflict of Interest Statement

The authors declare that the research was conducted in the absence of any commercial or financial relationships that could be construed as a potential conflict of interest.
